# Quantitative Detection of Active Vibrios Associated with White Plague Disease in *Mussismilia braziliensis* Corals

**DOI:** 10.3389/fmicb.2017.02272

**Published:** 2017-11-17

**Authors:** Luciane A. Chimetto Tonon, Janelle R. Thompson, Ana P. B. Moreira, Gizele D. Garcia, Kevin Penn, Rachelle Lim, Roberto G. S. Berlinck, Cristiane C. Thompson, Fabiano L. Thompson

**Affiliations:** ^1^Laboratory of Organic Chemistry of Biological Systems, Chemical Institute of São Carlos, University of São Paulo, São Carlos, Brazil; ^2^Civil and Environmental Engineering, Massachusetts Institute of Technology, Cambridge, MA, United States; ^3^Laboratory of Microbiology, Institute of Biology, SAGE-COPPE, Federal University of Rio de Janeiro, Rio de Janeiro, Brazil; ^4^Federal University of Rio de Janeiro (UFRJ), Rio de Janeiro, Brazil

**Keywords:** *Vibrio coralliilyticus*, *Mussismilia braziliensis*, *pyrH* gene, reef health monitoring, marine biology, biodiversity, microbiology

## Abstract

Over recent decades several coral diseases have been reported as a significant threat to coral reef ecosystems causing the decline of corals cover and diversity around the world. The development of techniques that improve the ability to detect and quantify microbial agents involved in coral disease will aid in the elucidation of disease cause, facilitating coral disease detection and diagnosis, identification and pathogen monitoring, pathogen sources, vectors, and reservoirs. The genus *Vibrio* is known to harbor pathogenic strains to marine organisms. One of the best-characterized coral pathogens is *Vibrio coralliilyticus*, an aetilogic agent of White Plague Disease (WPD). We used *Mussismilia* coral tissue (healthy and diseased specimens) to develop a rapid reproducible detection system for vibrios based on RT-QPCR and SYBR chemistry. We were able to detect total vibrios in expressed RNA targeting the 16S rRNA gene at 5.23 × 10^6^ copies/μg RNA and *V. coralliilyticus* targeting the *pyrH* gene at 5.10 × 10^3^ copies/μg RNA in coral tissue. Detection of *V. coralliilyticus* in diseased and in healthy samples suggests that WPD in the Abrolhos Bank may be caused by a consortium of microorganism and not only a single pathogen. We developed a more practical and economic system compared with probe uses for the real-time detection and quantification of vibrios from coral tissues by using the 16S rRNA and pyrH gene. This qPCR assay is a reliable tool for the monitoring of coral pathogens, and can be useful to prevent, control, or reduce impacts in this ecosystem.

## Introduction

Despite the undeniable importance of coral reefs around the World, they are undergoing massive extinction due to anthropogenic (e.g., overfishing and pollution) and global impacts (e.g., infectious diseases, ocean warming, and acidification; De'ath et al., 2009, 2012). A remarkable reduction in coral cover has been reported in both the Pacific and Caribbean reefs. In the south Atlantic, the coral reefs in Abrolhos constitute the most extensive and richest reefs in Brazilian waters (Leão and Kikuchi, 2001; Freitas et al., 2011; Osinga et al., 2011). *Mussismilia* species, the main Abrolhos reef builders, have suffered massively from white plague disease (WPD; Francini-Filho et al., 2010; McDole et al., 2012; Garcia et al., 2013). Corals affected by WPD show a pronounced line of bright, white tissue that separates the colored (living) part of the coral from bare, rapidly algal-colonized skeleton (Richardson et al., 2001). A range of different white diseases including WPD (Richardson et al., 1998a, 2001), white band (Aronson and Precht, 2001) and “shutdown reaction” (Antonius, 1981; Bythell et al., 2004) are widespread and collectively referred to as white syndromes (Bythell et al., 2004). On Indo-Pacific scleractinian corals a spreading band of tissue loss exposing the white skeleton is a symptom indicative for the classification of White Syndrome (WS; Willis et al., 2004).

*Vibrio coralliilyticus* has been identified as the causative agent of white plague disease in several Pacific reefs (Sussman et al., 2008, 2009). In hard corals, infection by *V. coralliilyticus* causes loss of *Symbiodinium* cells from the coenosarc tissue (the live tissue between polyps) and subsequent tissue loss exposing the white skeleton (Sussman et al., 2008, 2009). *V. coralliilyticus* P1 had high proteolytic activity as a result of the secretion of a set of proteases, including a Zinc-metalloprotease that plays an important role in the cleavage of connective tissue and other cellular perturbations (Santos Ede et al., 2011). *Vibrio harveyi* has also been considered as an important causal agent of WPD in tropical stony corals (Woodley et al., 2015). Luna et al. (2010), confirmed through Koch's postulates, the involvement of *V. harveyi* in the development of WPD. The inoculation of *V. harveyi* strains in healthy colonies of *Pocillopora damicornis* induced the disease and tissue lysis. *V. harveyi* and *V. coralliilyticus* are also pathogenic for many marine organisms, such as fishes (e.g., flounders, groupers, sharks, seabream, seabass, and turbots; Gauger et al., 2006), mollusks, and prawns (Nicolas et al., 2002; Alavandi et al., 2006).

Detection of vibrios is an important tool to understand disease ecology. PCR-based diagnostic methods are rapid, specific and sensitive for the detection of vibrios, and it is able to be applied in a management situation, monitoring specific pathogens (Goarant and Merien, 2006; Pollock et al., 2010; Wilson et al., 2013; Ahmed et al., 2015; Garrido-Maestu et al., 2016). The qPCR technologies are based on two categories of fluorescence chemistries (oligonucleotide-specific probes and intercalating dyes). The cost of oligonucleotide probe technologies, including TaqMan and Molecular Beacon, can be very high limiting wide adoption. Intercalating dye technologies, such as SYBR Green, fluoresce as they anneal to the double-stranded DNA (dsDNA) that is synthesized during PCR amplification. Intercalating dyes are less expensive and work with traditional PCR primer sets, negating the time and labor- intensive design of specific probes (Pollock et al., 2011). Several qPCR assays have been described for the detection of pathogenic vibrio species based in Taqman chemistry: targeting *Vibrio aestuarianus* (Saulnier et al., 2009; McCleary and Henshilwood, 2015), *V. harveyi* (Schikorski et al., 2013), *Vibrio tapetis* (Bidault et al., 2015), and *V. coralliilyticus*, targeting the *dnaJ* gene and employing seeded seawater and seeded coral tissue (Pollock et al., 2010). *V. parahaemolyticus, Vibrio vulnificus*, and *V. cholerae* were detected in Galician mussels by using multiplex qPCR (Garrido-Maestu et al., 2016). *Vibrio alginolyticus* was detected in shellfish and shrimp using SYBR Green I chemistry targeting the groEL (Ahmed et al., 2015). The use of SYBR is cheaper for reagents and equipment, but deserve attention to confirm specificity.

Coral associated microbial communities are very diverse (Wegley et al., 2007; Fernando et al., 2015). We have previously shown that a majority of bacterial OTUs have a low prevalence among individual colonies suggesting dynamic assemblages (Fernando et al., 2015) which may be influenced by populations introduced via the water or through ingestion of food particles (Thompson et al., 2014). Such populations may have variable levels of activity including populations that are inactive or dead due to stresses associated with the coral host and populations that are activity metabolizing and growing in the holobiont.

Recently, it has been introduced the idea that the coral meta-organism or holobiont hosts a microbiome with distinct microbial sub-communities, including (1) a ubiquitous and stable core microbiome (consisting of very few symbiotic host-selected microbiota), (2) a microbiome of spatially and/or regionally explicit core microbes each filling functional niches, and (3) a highly variable microbial community that is responsive to biotic and abiotic processes across spatial and temporal scales (Hernandez-Agreda et al., 2016; Sweet and Bulling, 2017). Many biotic and abiotic factors (e.g., algal competition, age of the colony, temperature, pH, nutrients, light, dissolved organic carbon, etc.) can affect the composition of the microbiome in corals (Bourne and Webster, 2013; Hernandez-Agreda et al., 2016; Sweet and Bulling, 2017). For instance, when the dynamic of the microbiome is disrupted in response to stress, pathogenic microbes, and their relationship with the “normal” microbiome of the organism, may influence or drive disease processes in the holobiont. This complex interaction of pathogenic microbes has been named as pathobiome (Vayssier-Taussat et al., 2014; Sweet and Bulling, 2017).

For better understanding of active fraction of the microbiota that may mediate symptoms associated with WS we chose to develop an RT-qPCR protocol. The 16S rRNA gene was selected to quantify total vibrios due to the availability of validated group-specific primers and the expectation that the abundance of 16S rRNA copies in extracted RNA would be indicative of metabolic activity and growth of the targeted group in coral tissue. Since several disease-causing vibrios are closely-related and difficult to resolve via the 16S rRNA gene sequence, we also developed species-specific PCR primers targeting the *pyrH* gene, which resolves species-level differences among the vibrios (Thompson et al., 2005). The *pyrH* gene encodes the housekeeping gene uridine monophosphate kinase (UMP kinase) that participates in pyrimidine biosynthesis catalyzing the conversion of UMP into UDP (Voet and Voet, 2004). This gene has been identified as a highly-expressed colonization factor in *Vibrio* species such as *V. vulnificus* and *V. harveyi* (Kim et al., 2003; Lee et al., 2007; Guerrero-Ferreira and Nishiguchi, 2010). *PyrH* expression by vibrios is expected during coral colonization. Therefore, we tested the hypotheses that (i) diseased corals have a higher proportion of active vibrio counts (detected by RT-qPCR) than healthy corals (Hp1), and; (ii) Diseased coral has more *Vibrio coralliilyticus* and/or *V. harveyi* than healthy corals (Hp2).

The aim of the present study was to develop a rapid and reproducible tool for the detection of vibrios, *V. coralliilyticus* and *V. harveyi* in coral tissue. We used the method to investigate the presence and activity of vibrio species, including the *V. coralliilyticus* and *V. harveyi* in healthy and diseased coral samples from the Abrolhos Bank. Diseased samples displayed symptoms corresponding to those described by Work and Aeby (2006) as typical of WPD, i.e., tissue loss, distinctly separated from intact tissue and revealing an intact skeleton.

Here we developed a practical and economic system for the real-time detection and quantification of vibrios from coral tissues by using the 16S rRNA and *pyrH* gene.

## Materials and methods

### Bacterial strains: culture conditions and genomic DNA extraction

For use as standards during assay development cultures of *Vibrio* strains including *V. coralliilyticus* (CAIM 616^T^; YB1^T^; LMG 21349; LMG 10953), *V. neptunius* (INCO17^T^; RFT5), *V. tubiashii* (LMG 10936^T^), *V. harveyi* (R-246; B-392)*, V. parahaemolyticus* (R-241)*, V. communis* (R-233^T^), and *V. campbellii* (HY01) were grown overnight in Marine Agar at 28°C. Total genomic DNA was extracted by using the Wizard Genomic DNA Kit (Promega, Madison, Wisconsin, USA) according to the manufacturer's instructions.

### Site description and coral collection

Abrolhos Bank (AB) is located in the southwestern Atlantic Ocean (SAO). Fragments of *M. braziliensis* (healthy and white plague diseased) were sampled in August 2011 and Februrary 2012 in Parcel dos Abrolhos (17°57′32.7″/ 38°30′20.3″), located off-shore (~70 km), inside the protected area Abrolhos Marine National Park. Four white plague (D1–D4) plus two healthy samples (H1–H2) were collected in winter (08/18/2011); and five white plague (D5–D9) plus three healthy samples (H3–H5) were collected in summer (02/29/2012; Table 1). Sampling was performed by scuba diving with a hammer and a chisel. Fragments were immediately stored in polypropylene tubes, identified, and frozen in liquid nitrogen.

**Table 1:** Sample data information used in this study.

**Sample number**	**Sampling place**	**Sample type**	**Status**	**Coral species**	**Sampling data**
D1	Abrolhos bank	RNA	Diseased (white plague)	*Mussismilia brasiliensis*	19/08/2011
D2					19/08/2011
D3					19/08/2011
D4					19/08/2011
D5	Parcel dos Abrolhos				29/02/2012
D6					29/02/2012
D7					29/02/2012
D8					29/02/2012
D9					29/02/2012
H1	Abrolhos bank		Healthy		19/08/2011
H2					19/08/2011
H3	Parcel dos Abrolhos				29/02/2012
H4					29/02/2012
H5					29/02/2012

### RNA extraction

The RNA was obtained by macerating 100 mg of coral tissue in small particles, until it turns to dust, in a crucible using pistillate and liquid N_2_. Trizol (1 mL) was added and mixed by vortexing for 2 s. Tubes were kept at room temperature for 5 min and then 200 uL of chloroform was added and the solution was mixed for 15 s by shaking hands. Tubes were incubated at room temperature for 3 min and then centrifuged at 2,000 rpm for 15 min. The clear phase was transferred to a clean tube and the purification step was performed with RNeasy® mini kit (Qiagen group) as of step 5 according to the manufacturer's protocols. The samples were kept on ice during extraction procedures.

With this protocol we obtained the average number of 23 μg of RNA (SE = ±9.4) per 100 mg of coral tissue. The samples were stored at −80°C. All samples were resuspended in water electrophoresed on 1% agarose gel (Figure S1), and quantified on a NanoDrop ND-1000 Spectrophotometer (NanoDrop Technologies, Wilmington, DE, USA) to evaluate quality and purity. All samples analyzed are listed (Table 1).

### cDNA synthesis

Reverse transcription was performed with the QuantiTect Reverse Transcription Kit—QIAGEN, according to the manufacturer's protocols with minor modifications. A DNAse step was performed and random pentadecamer primers were used to generate the initial cDNA strands (Stangegaard et al., 2006), which were quantified using a NanoDrop Spectrophotometer (NanoDrop Technologies, Wilmington, DE, USA).

### Primer design

Primers for *Vibrio* detection were designed in MEGA 5 (Tamura et al., 2011) against a multiple alignment of *Vibrio* sequences from public and personal databases (Table 2, Figure S2, Support Material 1). Conserved regions were selected and putative primer combinations were tested *in silico* by using the NCBI tool Primer-BLAST (http://www.ncbi.nlm.nih.gov/tools/primer-blast/). We used the reliable taxonomic marker *pyrH* as target, which has higher discriminatory power than the 16S rRNA gene and allows the distinction of closely related *Vibrio* species (Thompson et al., 2005; Sawabe et al., 2007). The best combination of primer pairs, indicated through *in silico* tests, were analyzed by PCR.

**Table 2:** Primer information.

**CODE**	**Sequence 5′-3′**	**Tm (°C)**	**Amplicon (bp)**	**Gene target**	**Phylogenetic target**
567F	GGC GTA AAG CGC ATG CAG GT	61.4	114	16S rRNA (Thompson et al., 2004)	*Vibrio* spp.
680R	GAA ATT CTA CCC CCC TCT ACA G	54.3			
Vc pyrH F	CAA CTG GGC AGA CGC AAT CCG TGA GT	64.8	166	*pyrH* (this study)	*V. coralliilyticus*
Vc pyrH R	CGT AAA TAC GCC ATC AAC TTT TGT C	54.9			
Vh pyrH F	CTT GCA ACG GTA ATG AAC GGT TTG GCA	61.9	171	*pyrH* (this study)	*V. harveyi*
Vh pyrH R	AGT ACC TGC AGA GAA GAT TAC CAC T	57.1			

Predicted amplicon size was verified through conventional PCR using the Amplitaq Gold 360 Master Mix (Applied Biosystems) in a total volume of 25 μL reaction and primers at 10 μM. The program was 95°C–5 min, 40 cycles (95°C–30 s, range 50°C up to 68°C–1 min, 72°C–1 min), and 72°C–5 min. DNA template of the targets *V. coralliilyticus* (CAIM 616^T^; YB1^T^; LMG 21349; LMG 10953), *V. harveyi* (R-246; B-392) and phylogenetically closely related *Vibrio* species, *V. communis* (R-233^T^), *V. parahaemolyticus* (R-241), *V. campbellii* (HY01), *V. neptunius* (INCO17^T^; RFT5), and *V. tubiashii* (LMG 10936^T^) were included in each reaction. Primer dimerization was checked in 1% agarose gel. Primer sequences selected for this study were Vc_pyrHF (5′- CAA CTG GGC AGA CGC AAT CCG TGA GT-3′) and Vc_pyrHR (5′-CGT AAA TAC GCC ATC AAC TTT TGT C-3′) to quantify *V. coralliilyticus* and Vh_pyrHF (5′- CTT GCA ACG GTA ATG AAC GGT TTG GCA-3′) and Vh_pyrHR (5′-AGT ACC TGC AGA GAA GAT TAC CAC T-3′) to quantify *V. harveyi*. In addition, 16S rRNA gene primer pair 567F (5′-GGC GTA AAG CGC ATG CAG GT-3′) and 680R (5′-GAA ATT CTA CCCCCC TCT ACA G-3′; Thompson et al., 2004) were applied to quantify the total number of vibrio sequences per sample (Table 2).

### Quantitative polymerase chain reaction (qPCR)

Total vibrios, *V. coralliilyticus* and *V. harveyi* were quantified by qPCR using primer pairs 567F-−680R, Vc_pyrHF—Vc_pyrHR and Vh_pyrHF—Vh_pyrHR, respectively, in a LightCycler_480 Real-Time PCR system with software v. 1.5.0 (Roche Applied Sciences, Indianapolis, IN, USA) for calculation of crossing point (Cp) values and melting temperature (T_*m*_) analysis. The correct size of the amplicons obtained (114-bp 16S rRNA, 166-bp *pyrH*, 171-bp *pyrH* gene fragments) was confirmed by 1% agarose gel electrophoresis. qPCR reaction mixtures consisted of 10 μL of KAPA SYBR_ FAST 2X Master Mix (KAPABIOSYSTEMS, Woburn, MA, USA), 10 μM of each primer and 1 μL of cDNA template (20 ng/uL). Amplification followed the manufacturer's instructions. Briefly, reactions were subjected to a preincubation step of 95°C for 3 min, followed by 50 cycles of 95°C for 10 s, 58°C (16S primers), 60°C (Vc-*pyrH* primers) or 62°C (Vh-*pyrH* primers) for 20 s and 72°C for 1 s. Each sample was analyzed in triplicate, and Cp values were examined after amplification to verify consistency (i.e., coefficient of variation ≤ 3%). To confirm the specificity of amplification, melting temperatures (T_*m*_) of sample amplicons were confirmed to be within two standard deviations of the mean T_*m*_ associated with qPCR standards at concentrations of 10^1^–10^7^ copies per qPCR (82.65°C ± SD 0.097). The fluorescence history, melting- curves, and peaks can be visualized in Figure S3.

Standards for qPCR were prepared by dilution of genomic DNA. Ten-fold serial dilutions of genomic DNA from *V. coralliilyticus, V. harveyi*, and total vibrio represented by *V. neptunius* were used to generate the standard curves (SCs; Figure S4). Genomic DNA concentration was measured by nanodrop method. Gene copy number was determined based on reported genome size and gene frequency within each genome. A genome size of 3.5 Mbp (*V. harveyi*) and 5.68 Mbp (*V. coralliilyticus*) were used, corresponding to a single copy of the *pyrH* gene. Moreover, a genome size of 5 Mbp (*V. neptunius*) was used, corresponding to 10 copies of the 16S rRNA gene.

For calculation of the standard curve Cp values were plotted against Log10 of computed gene copies/uL from genomic DNA added to each qPCR run using at least-squares fit. Confidence intervals for the predicted target concentrations based on measured Cp values were calculated based on the propagation of error in the SC (Harris, 1995). The limit of detection (LOD) was determined based on the uncertainty in the SC as the upper 99th per cent confidence interval of the Cp values of the negative controls or 50 cycles if no signal was apparent (Nshimyimana et al., 2014). For consistency in statistical analysis, the highest LOD used to indicate non-detectable target was selected as the study-wide LOD. The amplification efficiency (E) for each qPCR run was calculated from the slope of the standard curve and was considered consistent in the range of 89–100%. We determined the inhibition impact on qPCR by spiking the positive control DNA diluted 1:100, into an aliquot from each sample before qPCR amplification and by comparing the qPCR results measured for samples with and without spike addition. If the spiked sample was quantified as having <65% of the added amount of positive control (corresponding to both the 95% confidence interval for quantification of the qPCR standard curve and observed variability between technical replicates), then the sample was diluted 10-fold and re-analyzed (Nshimyimana et al., 2014).

To estimate the back-of-the-envelope conversion between copies per μg RNA and cells per ml, we used the average number of ribosomes per vibrio cell (that is 10) as determined by Lee et al. (2009), and the average number of μg of RNA (23 μg) extracted from 100 mg of coral tissue. Considering the density of coral tissue 1.15 g/mL (https://www.aqua-calc.com/page/density-table), which is close to the seawater 1.02 g/mL (https://www.aqua-calc.com/page/density-table/substance/seawater), we have 100 mg of coral tissue corresponds to 0.08 mL. So, in 1mL we found 287.5 μg RNA. Now, we have the formula: Cells/mL = {[n°qPCR copies/μgRNA x *x*(μgRNA/mL)] / *x*(n° of ribosomes vibrio cell)}.

To verify primer specificity in a closely-related non-target background target genomic DNA from *V. harveyi* or *V. coralliilyticus* were mixed with 10- to 100-fold excess of non-target DNA from *V. campbellii* (HY01), *V. communis* (R-233^T^), *V. neptunius* (INCO17^T^), *V. tubiashii* (LMG 10936^T^), or *E. coli*. All tested DNA were initially diluted to equimolar concentrations (100 ng/ul), then serial dilutions were performed and non-target DNA were kept 10-fold excess compared with target DNA mixed in the solution. Mixtures were quantified by QPCR as described above. If primers were specific for the target, then the observed concentrations would match the expected concentration of target DNA. In contrast, higher than expected observed concentrations would indicate non-specific amplification of closely related targets.

The percentage of the cross-reactivity specificity obtained from the target was calculated according to this equation:

$$\left(\frac{\text{qPCR copies of mixed }{\text{sample}}_{\left(\text{non-target }1/10\text{ plus target }1/100\right)\;}-\text{qPCR copies of non }-{\text{target}}_{\left(\text{non-target }1/10\right)\;}}{\text{qPCR copies of mixed }{\text{sample}}_{\left(\text{non-target }1/10\text{ plus target }1/100\right)}}\right)\times 100$$

### Statistical analyses

We tested the hypotheses that diseased corals have more total vibrios (Hp1) and more *V. coralliilyticus*/*V. harveyi* (Hp2) than health corals by Welch's *t*-test with the GraphPad software (https://www.graphpad.com/). We examined the interaction between health state and sampling season by two-way ANOVA using the OriginPro 8 SR0 software (Origin Lab Corporation). We tested the correlation between the abundance of all vibrios and the abundance of *V. coralliilyticus* using Spearman's Rho Correlation (http://www.socscistatistics.com/tests/Default.aspx).

## Results

### Primer specificity

The new primer pairs designed for the *pyrH* gene of *V. coralliilyticus* and *V. harveyi* showed specific match to the targeted species (Figures S5, S6). A single amplicon was observed when the target DNA was added whilst no amplification was observed when closely phylogenetic *Vibrio* species (i.e., *V. neptunius* INCO17^T^ and *V. tubiashii* LMG 10936^T^) were tested in standard PCR (Figure S5). This result confirmed the *in silico* selection of the primer pairs (Vc_pyrHF and Vc_pyrHR) for *V. coralliilyticus* LMG 20984^T^, CAIM 616^T^ (Accession Number GU266292). The same specificity was observed for the primer pairs Vh_pyrHF and Vh_pyrHR tested against *V. harveyi* R-246 (Accession Number EU251625), and closely related species *V. communis* (R-233^T^) and *V. parahaemolyticus* (R-241). Only the DNA fragment from the target could be amplified (171 bp) but not from the phylogenetic closely related species (Figure S6). Comparing the 16S rRNA sequence similarity among these *Vibrio* species we found that *V. coralliilyticus* shares up 98% of 16S rRNA sequence similarity with *V. neptunius* and *V. tubiashii*. while *V. harveyi* shares up 99% of 16S rRNA sequence similarity with *V. communis* and *V. parahaemolyticus*. The amplicons obtained for the genes tested were checked through sequencing and the specificity of this assay for the target strains was confirmed.

**Figure 1:**
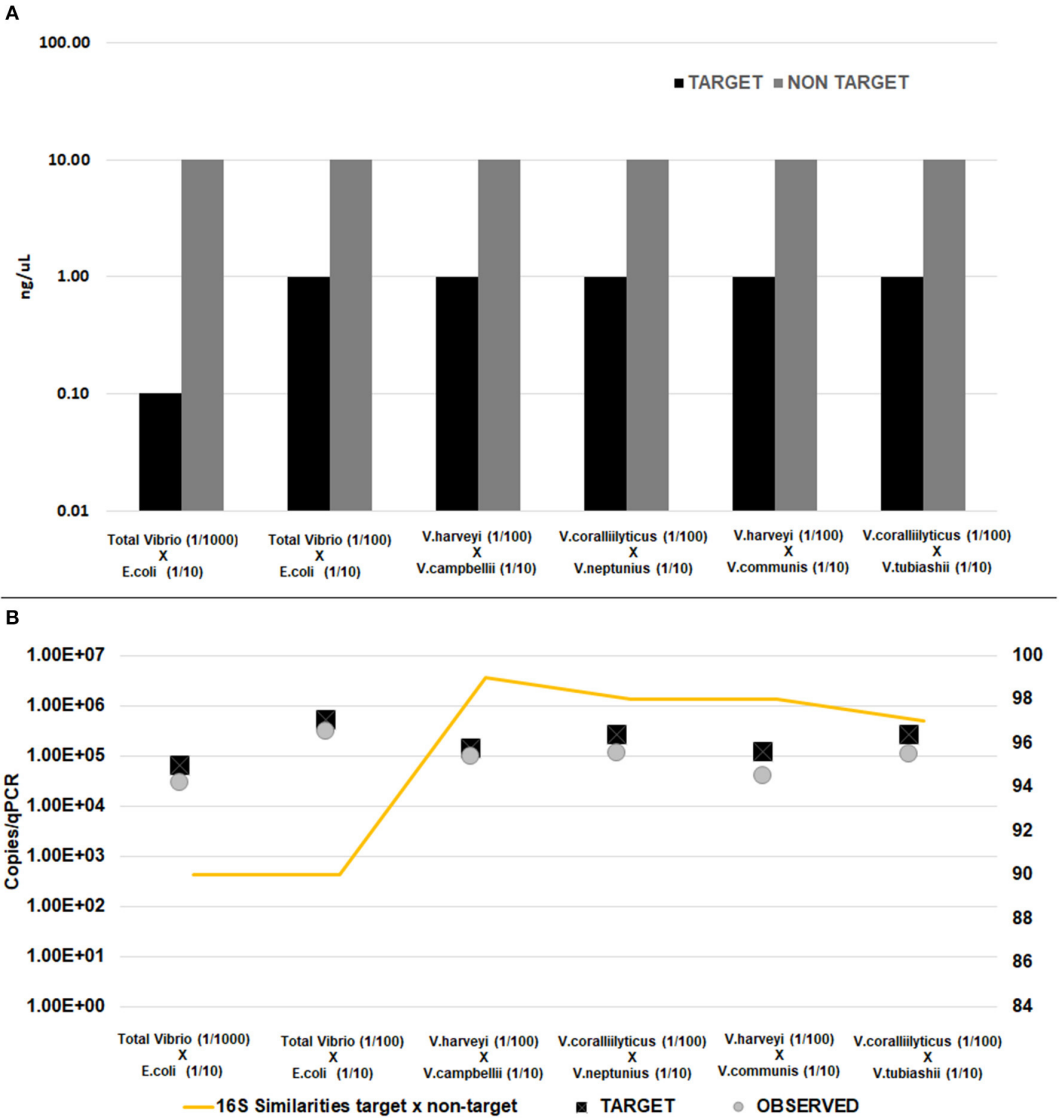
Amplification of target DNA in a background of closed related non-target. **(A)** Target and non-target DNA concentration used in the mixed reaction. **(B)** Copies detected by qPCR. All non-target DNA had 10-fold excess compare with target DNA. Target DNA i.e. (total *Vibrio, V. harveyi*, and *V. coralliilyticus*) were diluted at least 1:100 while non-target (i.e., *E. coli, V. campbellii, V. communis, V. tubiashii*, and *V. neptunius*) were diluted 1:10. Similarities of 16S rRNA between target and non-target are represented in line.

To determine the specificity of the assay in a background of closely-related non-target DNA qPCR analyses were performed on a mixture of non-target plus target DNA and up to 100% of the detection signal was due to the target DNA amplification. We observed that the values detected, represented basically only target DNA by qPCR (Figure 1). The number of copies obtained to the specific target species was very close to the observed in our assay. It means that the primers keep bind to the target species even when we have at least 10 times more concentrated non-target species phylogenetically closely related in the same sample. The percentage of the cross-reactivity specificity calculated showed that 100% of specificity was achieved when the target *V. harveyi* (1/100) was mixed with *V. communis* (1/10) and 80% when mixed with *V. campbellii* (1/10); 100% of the detection signal was achieved when the target *V. coralliilyticus* (1/100) was mixed with *V. tubiashii* (1/10) and 99% when mixed with *V. neptunius* (1/10); 99% when mixing *E. coli* (1/10) with *Vibrio* spp. (1/100) and 94% when *Vibrio* spp. (1/1,000) was mixed with *E. coli* (1/10).

### Total vibrios

**Figure 2:**
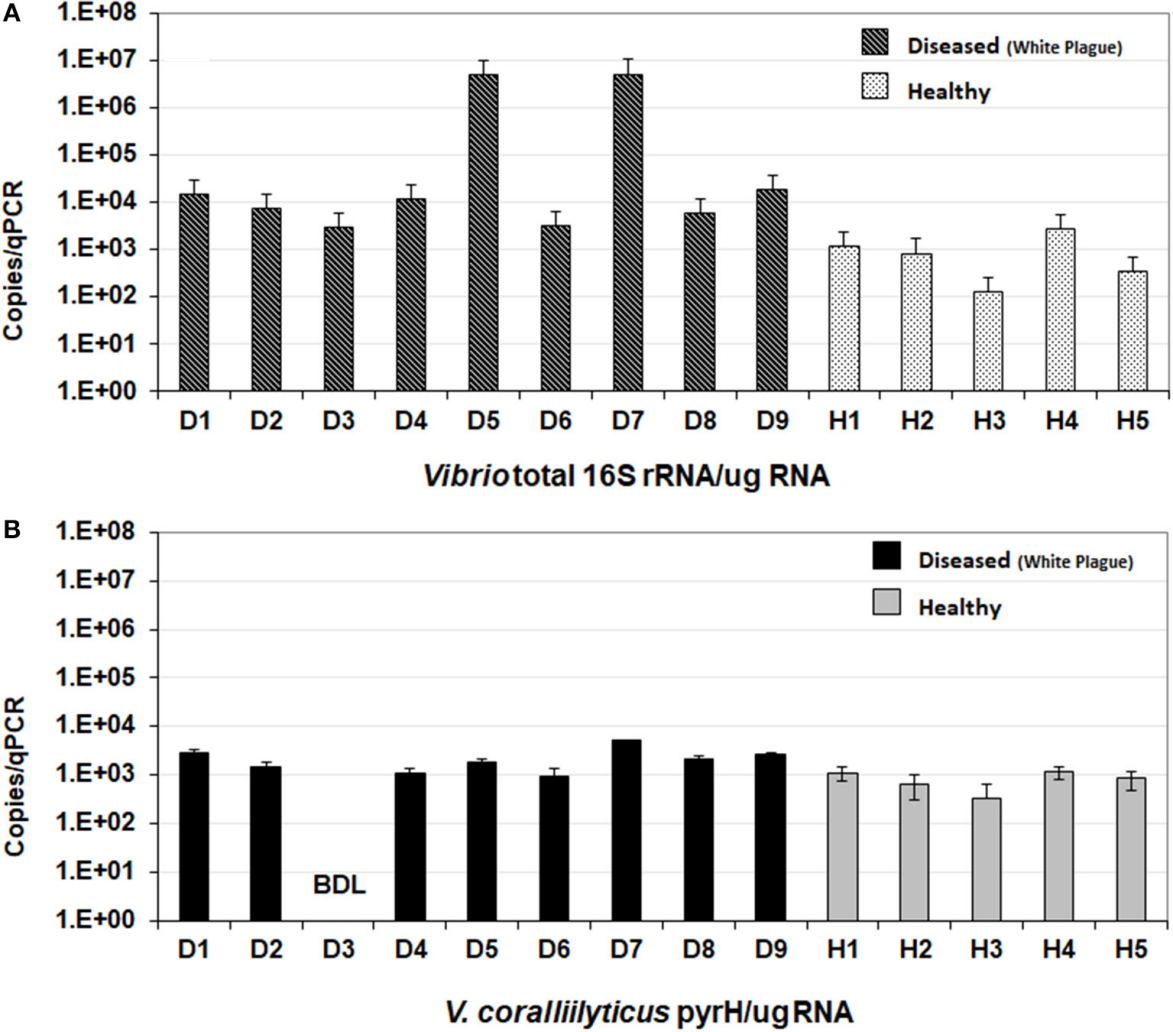
Detection of *Vibrio* species by qPCR approach in coral samples from Abrolhos region. **(A)** Number of total *Vibrio* specie detected by 16S rRNA gene amplification. **(B)** Number of *V. coralliilyticus* detected by pyrH gene amplification. W, White Plague; H, healthy; BDL, below detection limit.

Expressed *Vibrio* 16S rRNA gene varied from 134 to 2,750 copies per μg of RNA in healthy corals and from 3,000 to 5,230,000 per μg of RNA in diseased corals (Figure 2). Which represents the detection range of 3.85 × 10^3^ to 7.91 × 10^4^ cells/mL in health corals and 8.63 × 10^4^ to 1.5 × 10^8^ cells/mL in diseased corals. The load of total vibrios was significantly higher in diseased corals than in health corals (Welch's *t*-test, *p*-value = 0.0091). Two samples D5 and D7, collected during the summer sampling campaign showed over two orders of magnitude higher *Vibrio* RNA concentration compared to the other diseased or healthy corals. Nevertheless, the interaction between health state (health/disease) and sampling season (summer/winter) by two-way ANOVA was not significant.

#### *V. harveyi* and *V. coralliilyticus*

Expressed *V. harveyi* RNA was not detected in the coral samples by RT-qPCR of the *pyrH* gene. On the other hand, *V. coralliilyticus* was detected in all samples analyzed with exception of D3 (Figure 2). Expressed *V. coralliilyticus pyrH* gene varied from 658 to 1,160 copies per μg of RNA in healthy corals and from 973 to 5,100 per μg of RNA in diseased corals. The load of *V. coralliilyticus* was significantly higher in diseased corals than in health corals (Welch's *t*-test, *p* = 0.0194). However, the interaction between health state and sampling season by two-way ANOVA was not significant. Copy numbers of *V. coralliilyticus* and total *Vibrio* 16S rRNA were positively correlated (Spearman's Rho Correlation, *p* = 0.00056).

## Discussion

Quantification of *Vibrio* populations has been investigated in the last decades through culture-independent methods targeting DNA and whole cells (Eilers et al., 2000; Heidelberg et al., 2002; Thompson et al., 2004). For instance, Thompson and co-authors were able to quantify vibrios at 37 – 8 × 10^3^ cells/mL by quantitative PCR combined with constant denaturant capillary electrophoresis (qPCR-CDCE) of environmental DNA while methods based on detection of rRNA have quantified similar levels of individual cells via fluorescence *in situ* hybridization (FISH) followed by enumeration by microscopy of flow-cytometry (FCM). Eilers et al. (2000) detected 8 × 10^3^–1 × 10^4^ by using FISH and Heidelberg et al. (2002) detected vibrios 5 × 10^3^–1 × 10^5^ cells/mL by FCM. Here, we detected *Vibrio* 16S rRNAs at 3.85 × 10^3^–1.5 × 10^8^ cells/mL from environmental corals, showing a very rapid, specific, and sensitive tool for vibrios detection.

We suggest that both cell number and cell activity are important parameters for the investigation of coral disease. Kim et al. (2003) showed the importance of *pyrH* gene expression in *Vibrio*. Mutants unable to produce UMP kinase directly reducing bacterial growth and decreasing infectivity, revealing that *pyr*H expression has relevance in host colonization.

Luna et al. (2010) detected *V. harveyi* in tropical stony showing tissue necrosis and it was the most represented species recovered from diseased corals. Moreover, the inoculation of *V. harveyi* in healthy colonies of *P. damicornis* induced white plague disease. Surprisingly, we did not detect active *V. harveyi* in these samples. It is possible that *V. harveyi* is associated with the corals at a low level or is not actively expressing RNA due to a dormant like state or is not expressing *pyrH*. Further studies to compare diversity recovered by DNA and RNA-based methods will be necessary to shed further light on this.

*V. coralliilyticus* has been reported to have specific role in coral disease as a causative agent of WS in *Montipora aequituberculata, Pachyseris speciosa*, and *P. damicornis* corals (Sussman et al., 2008; Luna et al., 2010). In the present study *Vibrio corallilyticus* seems also been involved as one of causative agent of WPD in *Mussismilia*, which is reinforced by the statistical significance of Hp2. Active vibrios were more abundant in disease than heathy samples, with the highest vibrio activity detected in the two samples (D5 and D7) collected during summer months when disease outbreaks have been shown to be most prevalent (Figure 2; Francini-Filho et al., 2010). Active populations of *V. coralliilyticus* were detected in 13 of 14 coral sampled.

Although, in this study we found that diseased coral had higher vibrio counts than heathy ones and *V. corallilyticus* may have a role in WPD, it's still not clear if a consortium of vibrios instead of a single *Vibrio* species can produce WPD in *M. braziliensis* in the Abrolhos Bank. The idea that a collection or consortium of microbiota play a direct role in the causation of any given disease has recently been discussed in some studies as pathobiome concept (Vayssier-Taussat et al., 2014; Sweet and Bulling, 2017). The pathobiome breaks down the idea of “one pathogen = one disease” and highlights the role of certain members within the microbiome in causing pathogenesis. Moreover, studies of infectious agents have demonstrated that Koch's and Hill's fundamental postulates of “one microbe = one disease” has its limits (Vayssier-Taussat et al., 2014).

We note that *one* of the eight diseased corals tested did not reveal active *V. coralliilyticus* which may indicate that *V. coralliilyticus* is not necessary to establish the disease. However, we cannot rule out the possibility of multiple etiological agents for the same set of symptoms. At least three types of WPD has been described I (Dustan, 1977), II (Richardson et al., 1998a), and III (Richardson et al., 2001), differing in the rate of progression across a coral's surface and affect different species (Richardson et al., 2001; Sutherland et al., 2004). The literature reports three main bacteria specie as the causative pathogen involved in the WPD: *Sphingomonas* (Richardson et al., 1998b), *Aurantimonas coralicida* (Denner et al., 2003) in the Caribbean, and *Thalassomonas loyana* (Thompson et al., 2006) in the Red Sea, while additional agents of the similarly defined WS include several vibrios. Because no pathogen has been unequivocally verified as responsible for WPD, the debate regarding whether a definitive pathogen exists or whether different pathogens or bacterial consortia produce a similar disease phenotype in different coral species still remains (Roder et al., 2014a,b). Given the inherent difficulties of assigning a pathogen to WPD, in the Great Barrier Reef and Indo-Pacific region, WP-like phenotypes have been denominated WS (Willis et al., 2004).

## Conclusions

This study developed a reliable tool for the detection of active vibrios extensively tested with pure cultures and then with environmental samples from Abrolhos as a first attempt to disclose possible associations between vibrios and disease in *Mussismilia*.

It is important to highlight that the robustness of the RT-qPCR assay is supported by accurate quantification of target with the presence of competing non-*V. coralliilyticus* bacterial DNA which had a minimal impact on the target detection. Indeed, because the target organism is embedded within a matrix of other microbial and host cells in the holobiont, it is mandatory to establish accurate quantification in a background of complex targets to attain accurate detection. The real time PCR tool will be valuable for the detection of vibrios in corals as a possible management tool to foresee disease outbreaks.

The current study developed a practical and economic system for the real-time detection and quantification of vibrios from coral tissues. We were able to detect total vibrios using the 16S rRNA gene and *V. coralliilyticus* using the *pyrH* gene. With our protocols, the lowest limit detected for total vibrios and *V. coralliilyticus* from coral tissues of environmental samples was 134 and 658 copies/μg RNA, respectively. This qPCR assay allows the monitoring of samples naturally infected with *V. coralliilyticus* and may be useful for monitoring the health of coral reefs.

## Author contributions

LC and JT conceived and designed experiments. LC, KP, and RL carried out experiments and collected data, LC, JT, and AM performed data analysis. AM and GG contributed in field collection. LC wrote the paper. LC, JT, AM, RB, CT, and FT contributed with corrections and discussion. All authors contributed substantially to revisions.

### Conflict of interest statement

The authors declare that the research was conducted in the absence of any commercial or financial relationships that could be construed as a potential conflict of interest.
