# Supplementary material for: Quantitative Detection of Active Vibrios Associated with White Plague Disease in Mussismilia braziliensis Corals
Source: Front Microbiol. 2017 Nov 17;8:2272. doi: 10.3389/fmicb.2017.02272 (PMC5698304; doi:10.3389/fmicb.2017.02272)
Supplement: Supplementary file 1 [file DataSheet1.DOCX]

**Supplementary Material**

**Quantitative detection of active vibrios associated with white plague disease in *Mussismilia braziliensis* corals**

Luciane A. Chimetto Tonon^1,2,3*^, Janelle R. Thompson^2*^, Ana Paula B. Moreira^3^, Gizele D. Garcia^4^, Kevin Penn^2^, Rachelle Lim^2^, Roberto G. S. Berlinck^1^, Cristiane C. Thompson^3^, Fabiano L. Thompson^3^*

***Corresponding author**:

luciane.chimetto@gmail.com; [janelle@mit.edu](mailto:janelle@mit.edu); [fabianothompson1@gmail.com](mailto:fabianothompson1@gmail.com);

**Suplementary Figures**

**
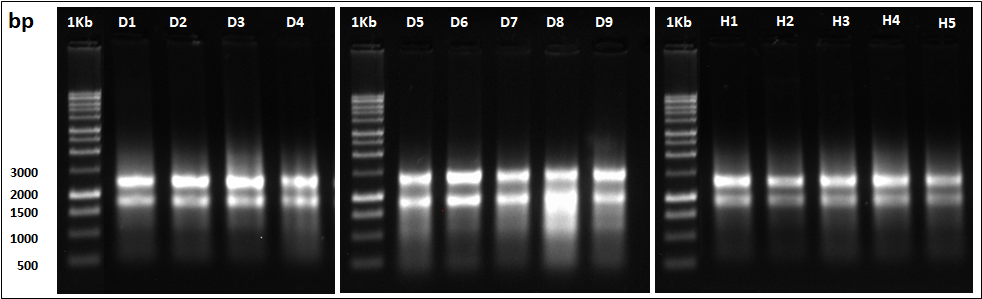
**

**Supplementary Figure S1**. RNA samples obtained from the coral *Mussismilia braziliensis* analyzed. H = Health sample. D= Diseased sample. (H1 - H2) and (D1 - D4) were collected in winter (08/18/2011); (D5 - D9) and (H3 - H5) were collected in summer (02/29/2012). The gel was performed with 1% agarose and SYBER® safe was used to stain the RNA samples. The Ladder (1Kb) is represented in the first line of each gel.


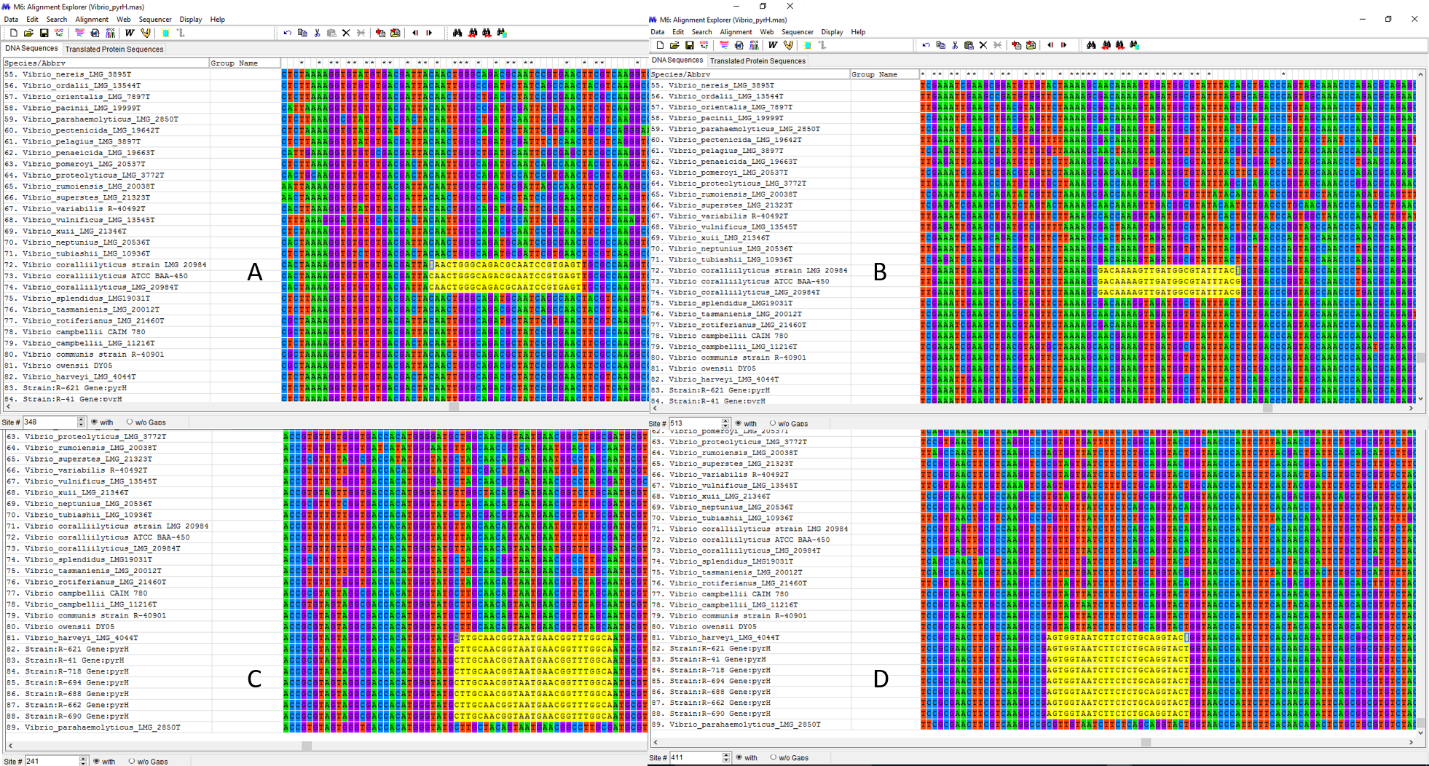


**Supplementary Figure S2.** Alignment of the Vibrio *pyrH* gene sequences showing the primer position and specificity against *V. coralliilyticus* and *V. harveyi* species. **A** = Vc_pyrHF, **B**= Vc_pyrHR, **C**= Vh_pyrHF and **D**= Vh_pyrHR

*
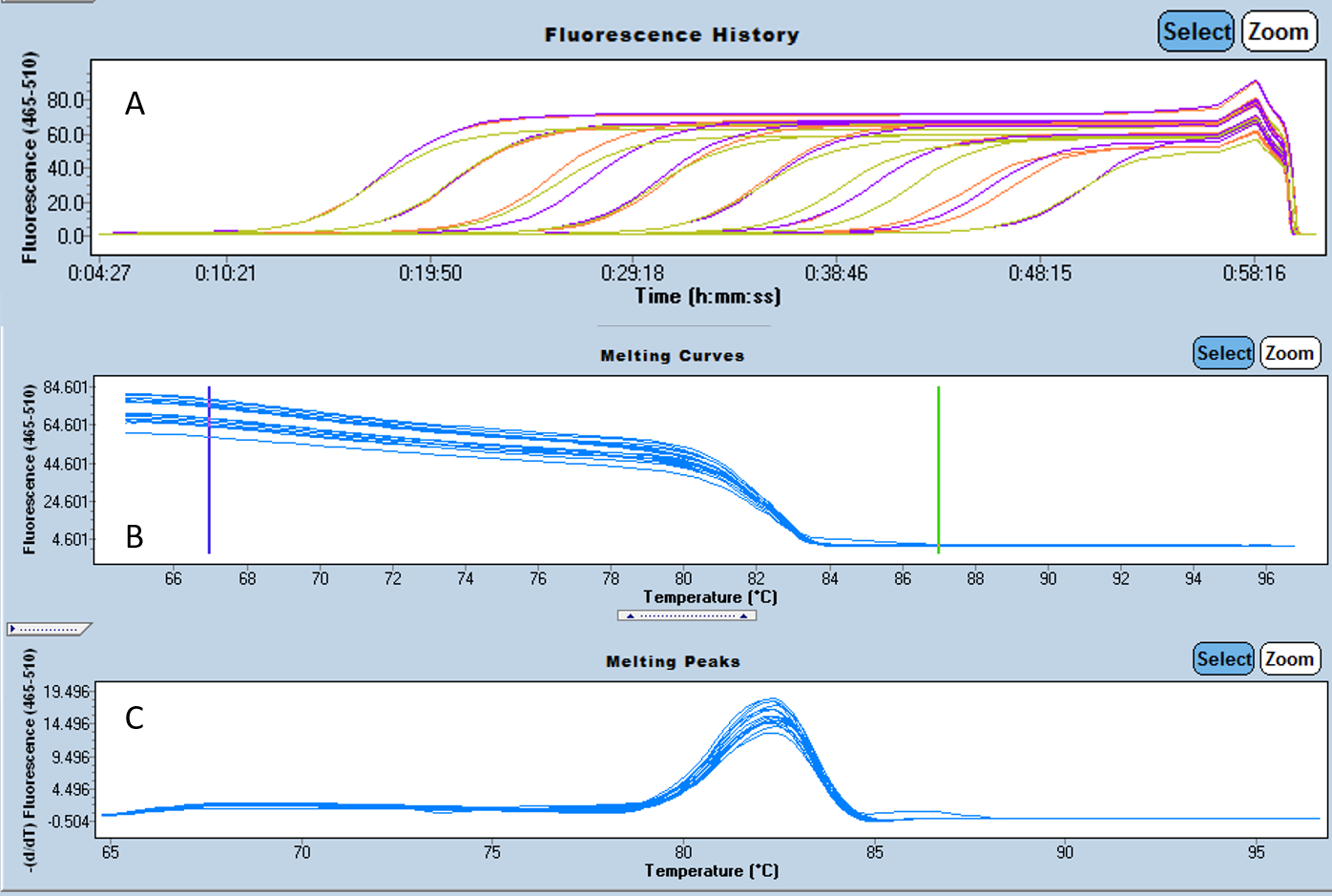
*

**Supplementary Figure S3**. Standard Curve. A= Florescence history through time run. B= Melting curves. C= Melting picks.


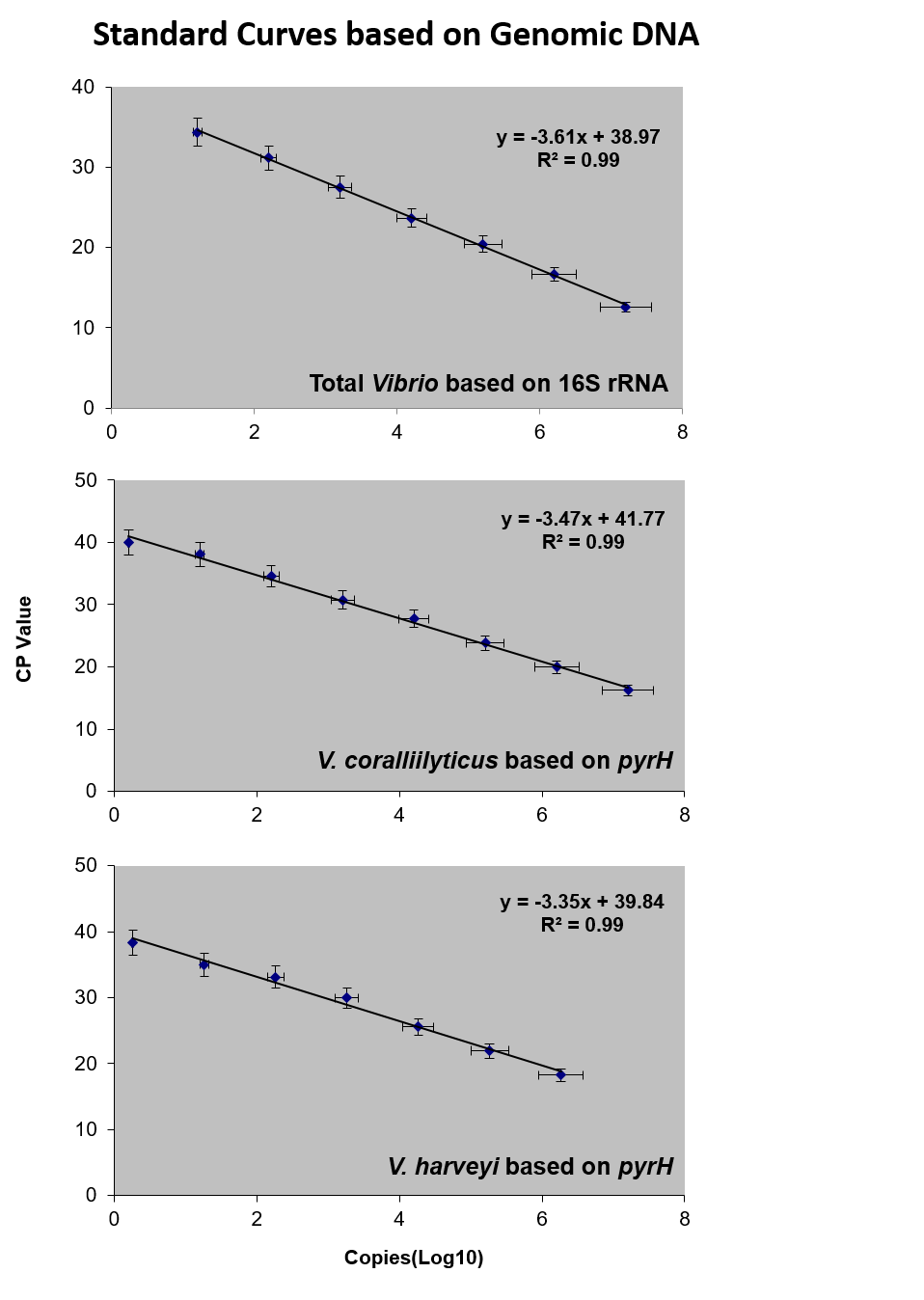


**Supplementary Figure S4.** Standard curve based on serial tenfold dilutions of DNA from total *Vibrio* (*Vibrio neptunius), V. coralliilyticus* and *V. harveyi.*

**
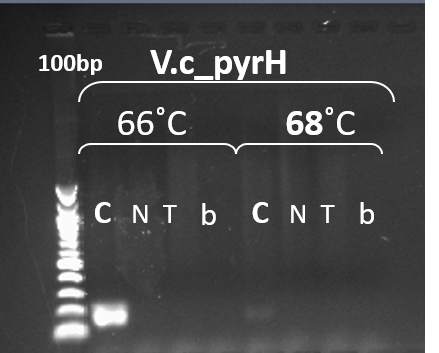
**

**Supplementary Figure S5**. Primer specificity. The electrophorese gel 1% agarose show the specific band at 166bp with the side of the amplicon obtained by *in silico* standard PCR using the primer pairs (Vc_pyrHF - Vc_pyrHR). **C** = *V. coralliilyticus*. **N** = *V. neptunius.* **T** = *V. tubiashii.* **b** = Blank.

*
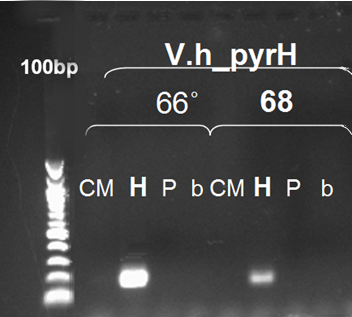
*

**Supplementary Figure S6**. Primer specificity. The electrophorese gel 1% agarose show the specific band at 171bp, which corresponds to the side of the amplicon obtained by *in silico* standard PCR using the primer pairs (Vh_pyrHF - Vh_pyrHR). **H** = *V. harveyi*. **CM** = *V. communis.* **P** = *V. parahaemolyticus.* **b** = Blank

**Support material 1**. Gene sequences of *pyrH* genes from strains used to design the primers.

>Vibrio campbellii_CAIM 780_FM202559

---------------CCAGCATATCAACGTATTCTATTAAAACTAAGTGGTGAAGCTCTTCAAGGTGAAGAAGGTTTTGGTATTGATCCAGCGGTTCTTGACCGTATGGCTCAAGAAGTGAAAGAGCTGGTTGAACTGGGTGTTCAAGTTGGTGTGGTAATCGGCGGTGGTAACTTGTTCCGTGGTGCAGGTCTTGCAGAAGCGGGTATGAACCGCGTAGTAGGCGACCACATGGGTATGCTTGCAACAGTAATGAACGGTCTAGCAATGCGTGATGCACTTCACCGTGCTTATGTAAACGCTCGCGTAATGTCTGCAATTCCGCTAAAAGGTGTGTGTGACGATTACAATTGGGCAGACGCTATCCGCGAACTTCGCCAAGGCCGTGTAGTTATCTTCTCTGCAGGTACTGGTAACCCATTCTTCACAACAGATTCAGCAGCGTGTCTACGCGGTATCGAAATCGAAGCTGACGTAGTTCTAAAAGCAACGAAAGTTGATGGTGTATTTACTGCTGACCCAGTAGCAAACCCAGACGCAGAGCTGTATGATAAGCTATCTTACACTGAAATTCTGGATAAAGAATTGAAAGTAATGGAC

>Vibrio_campbellii_LMG_11216T

--------------------------------------------------------------------------------------------------------------TCAAGAAGTGAAAGAGCTGGTTGAACTGGGTGTTCAAGTTGGTGTGGTAATCGGCGGTGGTAACCTGTTCCGTGGTGCTGGTCTTGCGGAAGCGGGTATGAACCGTGTAGTAGGCGACCACATGGGTATGCTTGCAACAGTAATGAACGGTCTAGCAATGCGTGATGCACTTCACCGTGCTTACGTAAACGCTCGTGTAATGTCTGCAATTCCTCTAAAAGGTGTGTGTGACGACTACAATTGGGCAGACGCTATCCGCGAACTTCGCCAAGGCCGTGTAGTAATCTTCTCTGCAGGTACTGGTAACCCATTCTTCACTACAGATTCAGCAGCGTGTCTACGTGGTATCGAAATCGAAGCTGACGTAGTGCTAAAAGCAACGAAAGTTGATGGCGTATTTACTGCTGACCCAGTAGCAAACCCAGATGCAGAGCTGTATGATAAGCTATCTTACAATGAAGTTCTGGATAAAGAACTGAAAGTAATGGACT---------------------------------------

>Vibrio harveyi ATCC:25919_EU130514

---------------------------------------------------------------------------------------------------------------CAAGAAGTGAAAGAGCTGGTTGAACTGGGTGTTCAAGTTGGTGTGGTAATCGGCGGTGGTAACCTGTTCCGTGGTGCAGGTCTTGCAGAAGCGGGTATGAACCGCGTAGTAGGCGACCACATGGGTATGCTTGCAACAGTAATGAACGGTCTAGCAATGCGTGACGCACTTCACCGTGCTTATGTAAACGCTCGCGTAATGTCTGCAATTCCGCTAAAAGGTGTGTGTGACGATTACAACTGGGCAGACGCTATCCGCGAACTTCGCCAAGGCCGTGTAGTTATCTTCTCTGCAGGTACTGGTAACCCATTCTTCACGACAGATTCAGCAGCGTGTCTACGCGGCATCGAAATCGAAGCTGACGTAGTTCTAAAAGCAACGAAAGTTGATGGTGTATTTACTGCTGACCCAGTAGCAAACCCAGACGCAGAGCTGTATGATAAGCTATCTTACACTGAAATTCTGGATAAAGAATTGAAAGTAATGGACTTGGCTGCGTTCACACTTGCT

>Vibrio_harveyi_LMG_4044T

-------------------------------------------------------------------------------------------------------------------------------------------------------------------------------------------------------AAGCTGGTATGAACCGCGTAGTAGGCGACCACATGGGTATGCTTGCAACGGTAATGAACGGTTTGGCAATGCGTGACGCACTTCACCGTGCATACGTAAACGCTCGTGTAATGTCTGCAATTCCTCTAAAAGGTGTGTGTGACGACTACAATTGGGCAGACGCTATCCGCGAACTTCGTCAAGGCCGAGTGGTAATCTTCTCTGCAGGTACTGGTAACCCATTCTTCACAACAGATTCAGCGGCGTGTCTACGTGGTATCGAAATTGAAGCTGACGTAGTTCTAAAAGCAACGAAAGTTGATGGCGTATTTACTGCAGACCCAGTAGCAAACCCAGACGCAGAGCTGTATGATAAGCTAT---------------------------------------------------------------------------------

>Vibrio_rotiferianus_LMG_21460T

--------------------------------------------------------------------------------------------------------------TCAAGAAGTTAAAGAGCTGGTTGAACTGGGTGTTCAAGTTGGTGTGGTAATCGGCGGCGGTAACCTATTCCGTGGTGCTGGTCTAGCTGAAGCAGGCATGAACCGTGTTGTGGGTGACCACATGGGTATGCTAGCAACAGTAATGAACGGTCTAGCAATGCGTGATGCACTTCATCGCGCATACGTAAACGCTCGTGTAATGTCTGCAATTCCGCTAAAAGGTGTGTGTGACGATTACAATTGGGCAGATGCTATTCGTGAACTTCGTCAAGGCCGTGTAGTGATCTTCTCTGCAGGTACAGGTAACCCATTCTTCACGACGGATTCAGCAGCTTGTCTACGTGGTATCGAAATCGAAGCTGACGTAGTTCTAAAAGCGACAAAAGTTGATGGTGTATTTACTGCTGACCCAGTAGCAAACCCAGACGCAGAGCTGTATGATAAGCTATCTTACGCTGAAATTCTGGATAAAGAATTGAAAGTAATG-------------------------------------------

>Vibrio owensii strain DY05|GU111253.1|

--------------------------------------------------------------------------------------------------------------TCAAGAAGTGAAAGAGCTGGTTGAACTGGGTGTTCAAGTTGGTGTGGTAATCGGCGGTGGTAACCTGTTCCGTGGTGCAGGTCTTGCAGAAGCGGGTATGAACCGCGTAGTAGGCGACCACATGGGTATGCTTGCAACAGTAATGAACGGTCTAGCAATGCGTGACGCACTTCACCGTGCTTATGTAAACGCTCGCGTAATGTCTGCAATTCCGCTAAAAGGTGTGTGTGACGATTACAACTGGGCAGACGCTATCCGCGAACTTCGCCAAGGCCGTGTAGTTATCTTCTCTGCAGGTACTGGTAACCCATTCTTCACTACAGATTCAGCAGCGTGTCTACGCGGTATCGAAATCGAAGCTGACGTAGTTCTAAAAGCAACGAAAGTTGATGGTGTATTTACTGCTGACCCAGTAGCAAACCCAGACGCAGAGCTGTATGATA---------------------------------------------------------------------------------------

>Vibrio communis strain R-40901|GU078692.1|

--------------------------------------------------------------------------------------------------------------TCAAGAAGTGAAAGAGCTGGTTGAACTGGGTGTTCAAGTTGGTGTGGTAATCGGCGGTGGTAACCTGTTCCGTGGTGCAGGTCTTGCAGAAGCGGGTATGAACCGCGTAGTAGGCGACCACATGGGTATGCTTGCAACAGTAATGAACGGTCTAGCAATGCGTGACGCACTTCACCGTGCTTATGTAAACGCTCGCGTAATGTCTGCAATTCCGCTAAAAGGTGTGTGTGACGATTACAACTGGGCAGACGCTATCCGCGAACTTCGCCAAGGCCGTGTAGTTATCTTCTCTGCAGGTACTGGTAACCCATTCTTCACAACAGATTCAGCAGCGTGTCTACGCGGTATCGAAATCGAAGCTGACGTAGTTCTAAAAGCAACGAAAGTTGATGGTGTATTTACTGCTGACCCAGTAGCAAACCCAGACGCAGAGCTGTATGATAAGCTATCTTACACTGAAATTCTGGATAAAGAATTGAAAGTAATGGA-----------------------------------------

>A10_pyrH

--------------------------------------------------------------------------------------------------------------TCAAGAAGTGAAAGAGCTGGTTGAACTGGGTGTTCAAGTTGGTGTGGTAATCGGCGGTGGTAACTTGTTCCGTGGTGCAGGTCTTGCAGAAGCGGGTATGAACCGCGTAGTAGGCGACCACATGGGTATGCTTGCAACAGTAATGAACGGTCTAGCAATGCGTGACGCACTTCACCGTGCTTATGTAAACGCTCGCGTAATGTCTGCAATTCCGCTAAAAGGTGTGTGTGACGATTACAATTGGGCAGACGCTATCCGCGAACTTCGCCAAGGCCGTGTAGTTATCTTCTCTGCAGGTACTGGTAACCCATTCTTCACTACAGATTCAGCAGCGTGTCTACGCGGTATCGAAATCGAAGCTGACGTAGTTCTAAAAGCAACGAAAGTTGATGGTGTATTTACTGCTGACCCAGTAGCAAACCCAGACGCAGAGCTGTATGATAAGCTATCTTACACTGAAATTCTGGATAAAGAATTGAAAGTAATGGACTTGGCTGCGTTCACACTTGCTCGTGAC-------------

>A11_pyrH

--------------------------------------------------------------------------------------------------------------TCAAGAAGTGAAAGAGCTGGTTGAACTGGGTGTTCAAGTTGGTGTGGTAATCGGCGGTGGTAACTTGTTCCGTGGTGCAGGTCTTGCAGAAGCGGGTATGAACCGCGTAGTAGGCGACCACATGGGTATGCTTGCAACAGTAATGAACGGTCTAGCAATGCGTGACGCACTTCACCGTGCTTATGTAAACGCTCGCGTAATGTCTGCAATTCCGCTAAAAGGTGTGTGTGACGATTACAATTGGGCAGACGCTATCCGCGAACTTCGCCAAGGCCGTGTAGTTATCTTCTCTGCAGGTACTGGTAACCCATTCTTCACTACAGATTCAGCAGCGTGTCTACGCGGTATCGAAATCGAAGCTGACGTAGTTCTAAAAGCAACGAAAGTTGATGGTGTATTTACTGCTGACCCAGTAGCAAACCCAGACGCAGAGCTGTATGATAAGCTATCTTACACTGAAATTCTGGATAAAGAATTGAAAGTAATGGACTTGGCTGCGTTCACACTTGCTCGTGAC-------------

>A12_pyrH

--------------------------------------------------------------------------------------------------------------TCAAGAAGTGAAAGAGCTGGTTGAACTGGGTGTTCAAGTTGGTGTGGTAATCGGCGGTGGTAACTTGTTCCGTGGTGCAGGTCTTGCAGAAGCGGGTATGAACCGCGTAGTAGGCGACCACATGGGTATGCTTGCAACAGTAATGAACGGTCTAGCAATGCGTGACGCACTTCACCGTGCTTATGTAAACGCTCGCGTAATGTCTGCAATTCCGCTAAAAGGTGTGTGTGACGATTACAATTGGGCAGACGCTATCCGCGAACTTCGCCAAGGCCGTGTAGTTATCTTCTCTGCAGGTACTGGTAACCCATTCTTCACTACAGATTCAGCAGCGTGTCTACGCGGTATCGAAATCGAAGCTGACGTAGTTCTAAAAGCAACGAAAGTTGATGGTGTATTTACTGCTGACCCAGTAGCAAACCCAGACGCAGAGCTGTATGATAAGCTATCTTACACTGAAATTCTGGATAAAGAATTGAAAGTAATGGACTTGGCTGCGTTCACACTTGCTCGTGAC-------------

>A2_80F_530R

---------------------------------------------------------------------------------------------------------------CAAGAAGTGAAAGAGCTGGTTGAACTGGGTGTTCAAGTTGGTGTGGTAATCGGCGGTGGTAACTTGTTCCGTGGTGCAGGTCTTGCAGAAGCGGGTATGAACCGCGTAGTAGGCGACCACATGGGTATGCTTGCAACAGTAATGAACGGTCTAGCAATGCGTGACGCACTTCACCGTGCTTATGTAAACGCTCGCGTAATGTCTGCAATTCCGCTAAAAGGTGTGTGTGACGATTACAATTGGGCAGACGCTATCCGCGAACTTCGCCAAGGCCGTGTAGTTATCTTCTCTGCAGGTACTGGTAACCCATTCTTCACTACAGATTCAGCAGCGTGTCTACGCGGCATCGAAATCGAAGCTGACGTAGTTCTAAAAGCAACTAAAGTTGATGGTGTATTTACTGCAGACCCAGTAGCAAACCCAGACGCAGAGCTGTATGATAAGCTATCTTACACTGAAATTCTGGATAAAGAATTGAAAGTAATGGACTTGGCTGCGTTCACACTTGCTCGTGAC-------------

>A3_80F_530R

--------------------------------------------------------------------------------------------------------------GCAAGAAGTGAAAGAGCTGGTTGAACTGGGTGTTCAAGTTGGTGTGGTAATCGGCGGTGGTAACTTGTTCCGTGGTGCAGGTCTTGCAGAAGCGGGTATGAACCGCGTAGTAGGCGACCACATGGGTATGCTTGCAACAGTAATGAACGGTCTAGCAATGCGTGACGCACTTCACCGTGCTTATGTAAACGCTCGCGTAATGTCTGCAATTCCGCTAAAAGGTGTGTGTGACGATTACAATTGGGCAGACGCTATCCGCGAACTTCGCCAAGGCCGTGTAGTTATCTTCTCTGCAGGTACTGGTAACCCATTCTTCACTACAGATTCAGCAGCGTGTCTACGCGGCATCGAAATCGAAGCTGACGTAGTTCTAAAAGCAACTAAAGTTGATGGTGTATTTACTGCAGACCCAGTAGCAAACCCAGACGCAGAGCTGTATGATAAGCTATCTTACACTGAAATTCTGGATAAAGAATTGAAAGTAATGGACTTGGCTGCGTTCACACTTGCTCGTGAC-------------

>A4_80F

----------------------------------------------------------------------------------------------------------------------------------------------------------------TCGGCGGTGGTAACTTGTTCCGTGGTGCAGGTCTTGCAGAAGCGGGTATGAACCGCGTAGTAGGCGACCACATGGGTATGCTTGCAACAGTAATGAACGGTCTAGCAATGCGTGACGCACTTCACCGTGCTTATGTAAACGCTCGCGTAATGTCTGCAATTCCGCTAAAAGGTGTGTGTGACGATTACAATTGGGCAGACGCTATCCGCGAACTTCGCCAAGGCCGTGTAGTTATCTTCTCTGCAGGTACTGGTAACCCATTCTTCACTACAGATTCAGCAGCGTGTCTACGCGGCATCGAAATCGAAGCTGACGTAGTTCTAAAAGCAACTAAAGTTGATGGTGTATTTACTGCAGACCCAGTAGCAAACCCAGACGCAGAGCTGTATGATAAGCTATCTTACACTGAAATTCTGGATAAAGAATTGAAAGTAATGGACTTGGCTGCGTTCACACTTGCTCGTGAC-------------

>A5_80F_530R

---------------------------------------------------------------------------------------------------------------CAAGAAGTGAAAGAGCTGGTTGAACTGGGTGTTCAAGTTGGTGTGGTAATCGGCGGTGGTAACTTGTTCCGTGGTGCAGGTCTTGCAGAAGCGGGTATGAACCGCGTAGTAGGCGACCACATGGGTATGCTTGCAACAGTAATGAACGGTCTAGCAATGCGTGACGCACTTCACCGTGCTTATGTAAACGCTCGCGTAATGTCTGCAATTCCGCTAAAAGGTGTGTGTGACGATTACAATTGGGCAGACGCTATCCGCGAACTTCGCCAAGGCCGTGTAGTTATCTTCTCTGCAGGTACTGGTAACCCATTCTTCACTACAGATTCAGCAGCGTGTCTACGCGGCATCGAAATCGAAGCTGACGTAGTTCTAAAAGCAACTAAAGTTGATGGTGTATTTACTGCAGACCCAGTAGCAAACCCAGACGCAGAGCTGTATGATAAGCTATCTTACACTGAAATTCTGGATAAAGAATTGAAAGTAATGGACTTGGCTGCGTTCACACTTGCTCGTGAC-------------

>A9_pyrH

--------------------------------------------------------------------------------------------------------------TCAAGAAGTGAAAGAGCTGGTTGAACTGGGTGTTCAAGTTGGTGTGGTAATCGGCGGTGGTAACCTGTTCCGTGGTGCAGGTCTTGCAGAAGCGGGTATGAACCGCGTAGTAGGCGACCACATGGGTATGCTTGCAACAGTAATGAACGGTCTAGCAATGCGTGACGCACTTCACCGTGCTTATGTAAACGCTCGCGTAATGTCTGCAATTCCGCTAAAAGGTGTGTGTGACGATTACAATTGGGCAGACGCTATCCGCGAACTTCGCCAAGGCCGTGTAGTTATCTTCTCTGCAGGTACTGGTAACCCATTCTTCACTACAGATTCAGCAGCGTGTCTACGCGGTATCGAAATCGAAGCTGACGTAGTTCTAAAAGCAACGAAAGTTGATGGTGTATTTACTGCTGACCCAGTAGCAAACCCAGACGCAGAGCTGTATGATAAGCTATCTTACACTGAAATTCTGGATAAAGAATTGAAAGTAATGGACTTGGCTGCGTTCACACTTGCTCGTGAC-------------

>Vibrio_shilonii_LMG19703

--------------------------------------------------------------------------------------------------------------ACAAGAGGTTAAAGAACTGGTTGAACTGGGTGTTCAAGTTGGTGTGGTGATTGGTGGTGGTAACTTGTTCCGTGGTGCAGGCCTTGCTGAAGCAGGTATGAACCGTGTAGTTGGTGATCACATGGGTATGTTAGCAACGGTTATGAATGGCCTAGCTATGCGTGATGCTCTTCACCGTGCGTATGTGAACGCTCGTGTTATGTCTGCTATCCCTCTTAAAGGTGTTTGTGACGATTACAACTGGGCTGATGCTATTCGCGAATTACGTCAAGGTCGCGTCGTAATTTTCTCTGCAGGTACTGGTAACCCATTCTTCACAACTGACTCTGCTGCGTGTCTTCGTGGTATCGAAATCGAAGCTGATGTTGTGCTTAAAGCAACAAAAGTAGATGGTGTTTTCACGGCTGATCCAGTGGCTAACCCTGATGCAGTTTTATGTGATAAGCTTTCTTACAGTGCAGTTCTAGAGAAAGAGCTGAAAGTAATGG------------------------------------------

>Contig_A-158

--------------------------------------------------------------------------------------------------------------TCAAGAAGTTAAAGAACTGGTTGAACTGGGTGTTCAAGTTGGTGTGGTCATTGGTGGTGGTAACTTGTTCCGTGGTGCAGGCCTTGCTGAAGCAGGTATGAACCGTGTAGTTGGTGATCACATGGGTATGTTAGCAACGGTTATGAATGGCCTAGCTATGCGTGATGCTCTTCACCGTGCGTATGTGAACGCTCGTGTTATGTCTGCTATCCCTCTTAAAGGTGTTTGTGACGATTACAACTGGGCTGATGCTATTCGCGAATTACGTCAAGGTCGCGTCGTAATTTTCTCTGCAGGTACTGGTAACCCATTCTTCACAACTGACTCTGCTGCGTGTCTTCGTGGTATCGAAATCGAAGCTGATGTTGTGCTTAAAGCAACAAAAGTAGATGGTGTTTTCACGGCTGATCCAGTGGCTAACCCTGATGCAGTTTTATGTGATAAGCTTTCTTACAGTGCAGTTCTAGAGAAAGAGCTGAAAGTAATGGACTTGGCGGCATTCACATTGGCTCGTGAC-------------

>Contig_A-165

--------------------------------------------------------------------------------------------------------------TCAAGAAGTTAAAGAACTGGTTGAACTGGGTGTTCAAGTTGGTGTGGTCATTGGTGGTGGTAACTTGTTCCGTGGTGCAGGCCTTGCTGAAGCAGGTATGAACCGTGTAGTTGGTGATCACATGGGTATGTTAGCAACGGTTATGAATGGCCTAGCTATGCGTGATGCTCTTCACCGTGCGTATGTGAACGCTCGTGTTATGTCTGCTATCCCTCTTAAAGGTGTTTGTGACGATTACAACTGGGCTGATGCTATTCGCGAATTACGTCAAGGTCGCGTCGTAATTTTCTCTGCAGGTACTGGTAACCCATTCTTCACAACTGACTCTGCTGCGTGTCTTCGTGGTATCGAAATCGAAGCTGATGTTGTGCTTAAAGCAACAAAAGTAGATGGTGTTTTCACGGCTGATCCAGTGGCTAACCCTGATGCAGTTTTATGTGATAAGCTTTCTTACAGTGCAGTTCTAGAGAAAGAGCTGAAAGTAATGGACTTGGCGGCATTCACATTGGCTCGTGAC-------------

>Contig_A-176

--------------------------------------------------------------------------------------------------------------TCAAGAAGTTAAAGAACTGGTTGAACTGGGTGTTCAAGTTGGTGTGGTCATTGGTGGTGGTAACTTGTTCCGTGGTGCAGGCCTTGCTGAAGCAGGTATGAACCGTGTAGTTGGTGATCACATGGGTATGTTAGCAACGGTTATGAATGGCCTAGCTATGCGTGATGCTCTTCACCGTGCGTATGTGAACGCTCGTGTTATGTCTGCTATCCCTCTTAAAGGTGTTTGTGACGATTACAACTGGGCTGATGCTATTCGCGAATTACGTCAAGGTCGCGTCGTAATTTTCTCTGCAGGTACTGGTAACCCATTCTTCACAACTGACTCTGCTGCGTGTCTTCGTGGTATCGAAATCGAAGCTGATGTTGTGCTTAAAGCAACAAAAGTAGATGGTGTTTTCACGGCTGATCCAGTGGCTAACCCTGATGCAGTTTTATGTGATAAGCTTTCTTACAGTGCAGTTCTAGAGAAAGAGCTGAAAGTAATGGACTTGGCGGCATTCACA-------------------------

>Contig_A-182

--------------------------------------------------------------------------------------------------------------TCAAGAAGTTAAAGAACTGGTTGAACTGGGTGTTCAAGTTGGTGTGGTCATTGGTGGTGGTAACTTGTTCCGTGGTGCAGGCCTTGCTGAAGCAGGTATGAACCGTGTAGTTGGTGATCACATGGGTATGTTAGCAACGGTTATGAATGGCCTAGCTATGCGTGATGCTCTTCACCGTGCGTATGTGAACGCTCGTGTTATGTCTGCTATCCCTCTTAAAGGTGTTTGTGACGATTACAACTGGGCTGATGCTATTCGCGAATTACGTCAAGGTCGCGTCGTAATTTTCTCTGCAGGTACTGGTAACCCATTCTTCACAACTGACTCTGCTGCGTGTCTTCGTGGTATCGAAATCGAAGCTGATGTTGTGCTTAAAGCAACAAAAGTAGATGGTGTTTTCACGGCTGATCCAGTGGCTAACCCTGATGCAGTTTTATGTGATAAGCTTTCTTACAGTGCAGTTCTAGAGAAAGAGCTGAAAGTAATGGACTTGGCGGCATTCACATTGGCTCGTGAC-------------

>Contig_A-184

--------------------------------------------------------------------------------------------------------------TCAAGAAGTTAAAGAACTGGTTGAACTGGGTGTTCAAGTTGGTGTGGTCATTGGTGGTGGTAACTTGTTCCGTGGTGCAGGCCTTGCTGAAGCAGGTATGAACCGTGTAGTTGGTGATCACATGGGTATGTTAGCAACGGTTATGAATGGCCTAGCTATGCGTGATGCTCTTCACCGTGCGTATGTGAACGCTCGTGTTATGTCTGCTATCCCTCTTAAAGGTGTTTGTGACGATTACAACTGGGCTGATGCTATTCGCGAATTACGTCAAGGTCGCGTCGTAATTTTCTCTGCAGGTACTGGTAACCCATTCTTCACAACTGACTCTGCTGCGTGTCTTCGTGGTATCGAAATCGAAGCTGATGTTGTGCTTAAAGCAACAAAAGTAGATGGTGTTTTCACGGCTGATCCAGTGGCTAACCCTGATGCAGTTTTATGTGATAAGCTTTCTTACAGTGCAGTTCTAGAGAAAGAGCTGAAAGTAATGGACTTGGCGGCATTCACATTGGCTCGTGAC-------------

>Contig_A-192

--------------------------------------------------------------------------------------------------------------TCAAGAAGTTAAAGAACTGGTTGAACTGGGTGTTCAAGTTGGTGTGGTCATTGGTGGTGGTAACTTGTTCCGTGGTGCAGGCCTTGCTGAAGCAGGTATGAACCGTGTAGTTGGTGATCACATGGGTATGTTAGCAACGGTTATGAATGGCCTAGCTATGCGTGATGCTCTTCACCGTGCGTATGTGAACGCTCGTGTTATGTCTGCTATCCCTCTTAAAGGTGTTTGTGACGATTACAACTGGGCTGATGCTATTCGCGAATTACGTCAAGGTCGCGTCGTAATTTTCTCTGCAGGTACTGGTAACCCATTCTTCACAACTGACTCTGCTGCGTGTCTTCGTGGTATCGAAATCGAAGCTGATGTTGTGCTTAAAGCAACAAAAGTAGATGGTGTTTTCACAGCTGATCCAGTGGCTAACCCTGATGCAGTTTTATGTGATAAGCTTTCTTACAGCGCAGTTCTAGACAAAGAGCTGAAAGTAATGGACTTGGCGGCATTCACATTGGCTCGTGAC-------------

>Contig_A-214

--------------------------------------------------------------------------------------------------------------TCAAGAAGTTAAAGAACTGGTTGAACTGGGTGTTCAAGTTGGTGTGGTCATTGGTGGTGGTAACTTGTTCCGTGGTGCAGGCCTTGCTGAAGCAGGTATGAACCGTGTAGTTGGTGATCACATGGGTATGTTAGCAACGGTTATGAATGGCCTAGCTATGCGTGATGCTCTTCACCGTGCGTATGTGAACGCTCGTGTTATGTYTGCTATCCCTCTTAAAGGTGTTTGTGACGATTACAACTGGGCTGATGCTATTCGCGAATTACGTCAAGGTCGCGTCGTAATTTTYTCTGCAGGTACTGGTAACCCATTCTTCACAACTGACTCTGCTGCGTGTCTTCGTGGTATCGAAATCGAAGCTGATGTTGTGCTTAAAGCAACAAAAGTAGATGGTGTTTTCACGGCTGATCCAGTGGCTAACCCTGATGCAGTTTTATGTGATAAGCTTTCTTACAGTGCAGTTCTAGAAAAAGAGCTGAAAGTAATGGACTTGGCGGCATTCACATTGGCTCGTGA--------------

>A224_80F_530R

--------------------------------------------------------------------------------------------------------------------------------------------------------------------------TAACTTGTTCCGTGGTGCAGGCCTTGCTGAAGCAGGTATGAACCGTGTAGTTGGTGATCACATGGGTATGTTAGCAACGGTTATGAATGGCCTAGCTATGCGTGATGCTCTTCACCGTGCGTATGTGAACGCTCGTGTTATGTCTGCTATCCCTCTTAAAGGTGTTTGTGACGATTACAACTGGGCTGATGCTATTCGCGAATTACGTCAAGGTCGCGTCGTAATTTTCTCTGCAGGTACTGGTAACCCATTCTTCACAACTGACTCTGCTGCGTGTCTTCGTGGTATCGAAATCGAAGCTGATGTTGTGCTTAAAGCAACAAAAGTAGATGGTGTTTTCACGGCTGATCCAGTGGCTAACCCTGATGCAGTTTTATGTGATAAGCTTTCTTACAGTGCAGTTCTAGAGAAAGA--------------------------------------------------------

>Vibrio_mediterranei_LMG16836_spain

--------------------------------------------------------------------------------------------------------------ACAAGAGGTTAAAGAACTGGTTGAACTGGGTGTTCAAGTTGGTGTAGTCATTGGTGGTGGTAACTTGTTCCGTGGTGCAGGCCTTGCTGAAGCAGGTATGAACCGTGTAGTTGGTGATCACATGGGTATGCTAGCAACGGTTATGAATGGCCTAGCTATGCGTGATGCTCTTCACCGCGCGTATGTGAATGCACGTGTTATGTCTGCTATCCCTCTTAAAGGTGTTTGCGACGATTACAACTGGGCTGATGCTATTCGTGAATTACGTCAAGGCCGCGTGGTGATTTTCTCAGCAGGTACTGGTAACCCATTCTTCACAACGGATTCTGCTGCATGTCTTCGTGGTATCGAAATCGAAGCTGATGTTGTGCTTAAAGCAACAAAAGTAGATGGTGTTTTCACGGCTGATCCAGTGGCTAACCCTGATGCAGTTTTATGTGATAAGCTTTCTTACAGTGCAGTTCTAGAGAAAGAGCTGAAAGTAATGG------------------------------------------

>R-14990_Mediterranean_sea_water

--------------------------------------------------------------------------------------------------------------ACAAGAGGTTAAAGAACTGGTTGAACTGGGTGTTCAAGTTGGTGTAGTCATTGGTGGTGGTAACTTGTTCCGTGGTGCAGGCCTTGCTGAAGCAGGTATGAACCGTGTAGTTGGTGATCACATGGGTATGCTAGCAACGGTTATGAATGGCCTAGCTATGCGTGATGCTCTTCACCGCGCGTATGTGAATGCACGTGTTATGTCTGCTATCCCTCTTAAAGGTGTTTGCGACGATTACAACTGGGCTGATGCTATTCGTGAATTACGTCAAGGCCGCGTGGTAATTTTCTCAGCAGGTACTGGTAACCCATTCTTCACAACGGATTCTGCTGCATGTCTTCGTGGTATCGAAATCGAAGCTGATGTTGTGCTTAAAGCAACAAAAGTAGATGGTGTTTTCACGGCTGATCCAGTGGCTAACCCTGATGCAGTTTTATGTGATAAGCTTTCTTACAGTGCAGTTCTAGAGAAAGAGCTGAAAGTAATGGA-----------------------------------------

>Vibrio_mediterranei_LMG11258T

--------------------------------------------------------------------------------------------------------------ACAAGAGGTTAAAGAACTGGTTGAACTGGGTGTTCAAGTTGGTGTAGTCATTGGTGGTGGTAACTTGTTCCGTGGTGCAGGCCTTGCTGAAGCAGGTATGAACCGTGTAGTTGGTGATCACATGGGTATGCTAGCAACGGTTATGAATGGCCTAGCTATGCGTGATGCTCTTCACCGCGCGTATGTGAATGCACGTGTTATGTCTGCTATCCCTCTTAAAGGTGTTTGCGACGATTACAACTGGGCTGATGCTATTCGTGAATTACGTCAAGGCCGCGTGGTAATTTTCTCAGCAGGTACTGGTAACCCATTCTTCACAACGGATTCTGCTGCATGTCTTCGTGGTATCGAAATCGAAGCTGATGTTGTGCTTAAAGCAACAAAAGTAGATGGTGTTTTCACGGCTGATCCAGTGGCTAACCCTGATGCAGTTTTATGTGATAAGCTTTCTTACAGTGCAGTTCTAGAGA------------------------------------------------------------

>R-14988_Scophthalmus_maximus_larvae_gut_Belgium

--------------------------------------------------------------------------------------------------------------ACAAGAGGTTAAAGAACTGGTTGAACTGGGTGTTCAAGTTGGTGTAGTCATTGGTGGTGGTAACTTGTTCCGTGGTGCAGGCCTTGCTGAAGCAGGTATGAACCGTGTAGTTGGTGATCACATGGGTATGCTAGCAACGGTTATGAATGGCCTAGCTATGCGTGATGCTCTTCACCGCGCGTATGTGAATGCACGTGTTATGTCTGCTATCCCTCTTAAAGGTGTTTGCGACGATTACAACTGGGCTGATGCTATTCGTGAATTACGTCAAGGCCGCGTGGTGATTTTCTCAGCAGGTACTGGTAACCCATTCTTCACAACGGATTCTGCTGCATGTCTTCGTGGTATCGAAATCGAAGCTGATGTTGTGCTTAAAGCAACAAAAGTAGATGGTGTTTTCACGGCTGATCCAGTGGCTAACCCTGATGCAGTTTTATGTGATAAGCTTTCTTACAGTGCAGTTCT-----------------------------------------------------------------

>R-14989_rotifer_culture_water_Gent_Belgium

--------------------------------------------------------------------------------------------------------------ACAAGAGGTTAAAGAACTGGTTGAACTGGGTGTTCAAGTTGGTGTAGTCATTGGTGGTGGTAACTTGTTCCGTGGTGCAGGCCTTGCTGAAGCAGGTATGAACCGTGTAGTTGGTGATCACATGGGTATGCTAGCAACGGTTATGAATGGCCTAGCTATGCGTGATGCTCTTCACCGCGCGTATGTGAATGCACGTGTTATGTCTGCTATCCCTCTTAAAGGTGTTTGCGACGATTACAACTGGGCTGATGCTATTCGTGAATTACGTCAAGGCCGCGTGGTGATTTTCTCAGCAGGTACTGGTAACCCATTCTTCACAACGGATTCTGCTGCATGTCTTCGTGGTATCGAAATCGAAGCTGATGTTGTGCTTAAAGCAACAAAAGTAGATGGTGTTTTCACGGCTGATCCAGTGGCTAACCCTGATGCAGTTTTATGTGATAAGCTTTCTTACAGTGCAGT--------------------------------------------------------------------

>Vibrio breoganii strain RD 15.11

---------------------------------------------------------------------------TTTGGTATCGACCCAACTATCCTAGAGCGTATGGCTCAGGAAGTGAAAGAGTTAGTTGAACTAGGTGTAGAAGTTGGTGTGGTTATCGGTGGTGGTAACTTGTTCCGCGGTGCTGGCCTTGCAGAAGCAGGCATGAACCGCGTAGTTGGCGACCACATGGGTATGCTAGCAACAGTGATGAATGGCCTAGCTATGCGCGATGCACTGCACCGTGCATACGTGAATGCTCGCGTAATGTCAGCGATACAGCTTAAAGGCGTGTGTGACGATTACAACTGGGCTGACGCTATCCGCGAACTTCGCCAAGGTCGCGTAGTGATCTTCTCTGCAGGAACGGGTAACCCATTCTTCACAACGGACTCTGCTGCTTGTCTTCGTGGTATCGAGATCGAAGCGGATCTAGTACTAAAAGCAACAAAAGTTGACGGCGTATACAATGCTGACCCTGCAACGAACCCAGACGCTGAACTTTA--------------------------------------------------------------------------------------------

>Vibio_nigripulchritudo_LMG_3896T

--------------------------------------------------------------------------------------------------------------TCAAGAAGTAAAAGAACTGGTTGAACTGGGTGTTCAAGTGGGCGTGGTTATCGGTGGTGGTAACTTGTTCCGTGGTGCAGGTCTGGCTGAAGCGGGTATGAACCGCGTTGTGGGTGACCACATGGGTATGTTGGCAACCGTAATGAACGGCCTGGCAATGCGCGATGCGCTTCACCGTGCATACGTGAATGCTCGCGTTATGTCTGCAATTCCTCTAAAAGGTGTATGTGACGACTACAACTGGGCAGATGCGATTCGCGAACTGCGTCAAGGCCGTGTGGTTATCTTCTCTGCAGGTACAGGTAATCCATTCTTTACTACTGATTCTGCTGCTTGTTTGCGCGGTATTGAAATCGAAGCGGATGTAGTTCTAAAAGCAACAAAAGTAGATGGTGTATTTACTGCTGATCCGGTGGCAAACCCTGAAGCAGAGCTGTATGATACTCTTTCTTTTAGTGAAGTTCTTGAAAAAGAATTGAAAGTAA---------------------------------------------

>Photobacterium_phosphoreum_ATCC_11040T

--------------------------------------------------------------------------------------------------------------ACAAGAAGTAAAAGAACTCGTTGAATTAGGTGTTCAAGTCGGCCTTGTTATCGGAGGCGGTAACCTATTCCGTGGTGCTGGTCTGGCTGAAGCAGGTATGAACCGAGTTGTGGGCGACCACATGGGTATGCTAGCAACAGTAATGAACGGCTTAGCAATGCGTGATGCGCTGCACCGTGCCTATGTGAACGCGCGAGTAATGTCTGCTATTCCTCTAAACGGTGTATGTGATAATTATAACTGGGCTGATGCTATCAGTCAGTTACGTCAAGGCCGTGTTGTTATTTTCTCTGCAGGTACAGGCAATCCGTTCTTCACAACCGATTCTGCTGCATGTCTTCGTGGTATCGAAATCGAAGCTGATGTAGTATTGAAAGCAACAAAAGTTGAAGGCGTGTACACGGATGATCCAGTTAAAAATCCAGATGCTACGCTTTGTGAACAGCTGAGCTACCAAGATGTACTTGAAAAAGAACTTAAGGTCATGGATTTGGCTGCATTTACTCTTGCT-------------------

>Vibrio_aerogenes_LMG_19650T

--------------------------------------------------------------------------------------------------------------TCAGGAAGTAAAAGAGTTAGTTGAGCTGGGTGTTCAGGTTGGCGTCGTAATTGGTGGCGGCAACTTATTCCGTGGAGCCGGGCTGGCCAAAGCCGGGATGAATCGTGTGGTCGGTGACCATATGGGTATGCTGGCAACCGTGATGAATGGCCTTGCAATGCGTGACGCTCTTCACCGCGCTTATGTGAATGCCAGAGTGATGTCTGCGATCCCTTTGAATGGTGTTTGTGATGATTACAACTGGGCAAATGCGATCAGTCAGCTACGCCAGGGGCGTGTTGTGATTTTTTCGGCTGGCACCGGAAATCCATTTTTTACAACTGATTCTGCAGCTTGTTTACGTGGTATTGAAATTGAAGCGGATGTAGTTCTCAAAGCGACAAAAGTTGACGGAGTTTACAGTGCCGATCCGGTGGCAAACCCTGAGGCGGAGTTGTATGATAAGCTGAATTATGAAGATGTGCTCGAAAAAGAATTGAAAGTGATG-------------------------------------------

>Vibrio_aestuarianus_LMG_7909T

--------------------------------------------------------------------------------------------------------------TCAGGAAGTAAAAGAACTGGTTGAACTGGGTGTTCAAGTTGGCGTTGTTATCGGTGGTGGTAACTTATTCCGTGGTGCTGGTCTAGCTGAAGCTGGCATGAACCGTGTTGTTGGCGACCATATGGGTATGCTTGCGACAGTTATGAACGGCTTAGCAATGCGTGATGCTCTGCACCGTGCTTACGTGAACGCTCGTGTGATGTCGGCTATTCCTCTTAAAGGCGTTTGTGATGATTACAACTGGGCTGATGCGATTAGTCAATTGCGTCAAGGTCGAGTGGTTATTTTCTCAGCAGGTACTGGAAACCCATTCTTCACCACTGATTCTGCTGCATGTTTGCGCGGTATTGAGATTGAAGCAGACGTAGTTCTAAAAGCGACAAAAGTAGATGGCGTATTTAGTGCAGACCCAGTGGCAAACCCTGACGCAGAGCTGTATGATAAGCTGTCTTATACAACTGTGCTTGAAAAAGAGCTAAAAGTTATGGACGGGCCCCCCCTTCA--------------------------

>Vibrio_alginolyticus_LMG_4409T

--------------------------------------------------------------------------------------------------------------ACAAGAAGTTAAAGAACTGGTTGAACTGGGTGTTCAGGTTGGTGTAGTTATCGGTGGCGGTAACTTGTTCCGTGGTGCAGGCCTAGCTGAAGCTGGTATGAACCGCGTAGTTGGTGACCACATGGGTATGCTTGCTACAGTAATGAATGGCCTAGCGATGCGTGATGCTCTTCACCGTGCATACGTAAATGCGCGCGTTATGTCTGCAATCCCTCTAAAAGGCGTATGTGACGACTATAATTGGGCTGATGCGATCCGTGAGCTTCGCCAAGGCCGAGTTGTCATCTTCTCTGCTGGTACTGGTAACCCGTTCTTTACAACGGATTCTGCTGCTTGTTTACGTGGTATCGAAATTGAAGCGGATGTTGTACTAAAAGCGACGAAAGTTGATGGTGTATTTACTGCTGACCCTGTAGCAAACCCAGACGCAGAGCTGTATGATAAGCTATCTTATGCAGAAGTTCTGGATAAAGAGCTGAAAGTTATGGACTGGG------------------------------------

>Vibrio_anguillarum_LMG_4437T

--------------------------------------------------------------------------------------------------------------ACAGGAAGTAAAAGAACTGGTTGAACTGGGTGTTCAAGTGGGTGTTGTCATCGGCGGCGGTAACTTATTCCGTGGCGCAGGTCTTGCTGAAGCGGGCATGAACCGTGTTGTGGGCGACCACATGGGAATGCTGGCAACCGTAATGAATGGTTTGGCAATGCGTGATGCTTTGCACCGTGCTTATGTGAATGCGCGCGTGATGTCTGCCATTCCTCTTAAAGGTGTGTGTGACGATTACAATTGGGCCGATGCTATCAGCCAACTACGTCAAGGCCGTGTTGTGATCTTCTCAGCGGGTACTGGTAACCCATTCTTCACAACTGATTCTGCTGCCTGTTTGCGCGGTATTGAAATTGAAGCGGATGTGGTTCTAAAAGCGACAAAAGTAGATGGCGTATTTAGTGCAGACCCAGTGGCAAACCCAGACGCAGAACTGTATGATAAGCTGTCTTATACAACCGTGCTTGAAAAAGAGCTAAAAGTGATGGACCTGG------------------------------------

>Vibrio_brasiliensis_LMG_20546T

--------------------------------------------------------------------------------------------------------------TCAAGAAGTAAAAGAGTTGGTTGAACTTGGCGTTCAAGTTGGTGTAGTTATCGGTGGTGGTAACCTATTCCGTGGTGCTGGCCTTGCTGAAGCAGGTATGAACCGCGTTGTTGGTGACCACATGGGTATGCTAGCAACCGTAATGAACGGCCTTGCAATGCGTGACGCTCTACACCGTGCGTACGTAAATGCACGTGTAATGTCTGCAATTCCACTTAAAGGTGTGTGTGACGACTACAACTGGGCTGACGCAATCCGCGAACTACGCCAAGGTCGTGTAGTCATCTTCTCAGCGGGTACTGGTAACCCATTCTTCACTACAGATTCTGCTGCATGTTTGCGCGGTATCGAGATTGAAGCTGACGTAGTTCTAAAAGCGACAAAAGTTGATGGTGTATTTACTGCAGACCCAGTGGCAAACCCTGACGCAGAACTGTATGATAAGCTTTCATTCAATGCTGTTCTTGAAAAAGAACTGAAAGTGATGGA-----------------------------------------

>Vibrio_chagasii_LMG_21353T

--------------------------------------------------------------------------------------------------------------TCAAGAAGTAAAAGAATTGGTTGAACTAGGCGTTCAAGTTGGTGTTGTTATCGGTGGCGGTAACCTGTTCCGTGGTGCTGGTCTTGCTGAAGCTGGCATGAACCGCGTTGTTGGCGACCACATGGGTATGCTTGCAACAGTAATGAACGGCCTTGCTATGCGTGATGCTCTGCACCGTGCTTACGTAAACGCACGTGTAATGTCTGCAATTCCTCTAAAAGGCGTGTGTGACGACTACAACTGGGCAGATGCAATCAGCCAACTACGTCAAGGTCGCGTTGTGATCTTCTCTGCTGGTACTGGTAACCCATTCTTCACTACTGACTCTGCTGCATGTCTACGTGGTATCGAAATCGAAGCTGACGTAGTTCTAAAAGCGACAAAAGTAGATGGTGTATTTACTGCTGACCCAGTAGCAAACCCAGACGCAGAGCTGTATGATACTTTGTCTTACAACACAGTTCTTGAGAAAGAGCTTAAAGTAATGGACTGGG------------------------------------

>Vibrio_cholerae_LMG_21698T

--------------------------------------------------------------------------------------------------------------TCAAGAAGTAAAAGAACTGGTTGAGCTGGGTGTTCAAGTGGGCGTTGTGATTGGTGGCGGCAACCTATTCCGTGGTGCTGGCCTTGCCAAAGCGGGTATGAACCGTGTTGTTGGGGATCATATGGGCATGCTTGCCACGGTAATGAATGGTTTGGCAATGCGTGATGCCCTGCATCGTGCTTACGTCAATGCACGTCTGATGTCTGCTATCCCACTAAATGGTGTGTGTGACGATTACAGCTGGTCTGATGCGATTCGCGAATTGCGCCAAGGCCGAGTGGTGATTTTTGCTGCGGGTACAGGCAATCCTTTCTTTACTACGGATTCGGCTGCGTGTCTGCGTGGTATTGAAATTGAAGCTGATGTCGTTCTCAAAGCGACCAAAGTAGATGGTGTTTACAGCGCTGACCCGGTAGCCAACCCGGATGCACAACTGTATGATAAGCTGGCTTACAACGATGTGCTTGAGAAAGAACTGAAAGTGATGGACGGGCCCCCCCTTCA--------------------------

>Vibrio_cincinnatiensis_LMG_7891T

--------------------------------------------------------------------------------------------------------------ACAGGAAGTGAAAGAACTCGTGGAATTGGGGGTTCAAGTGGGGGTCGTCATTGGTGGCGGTAACTTGTTCCGAGGTGCAGGCCTTGCTTCTGCTGGCATGAACCGTGTTGTGGGTGACCATATGGGGATGTTGGCAACCGTGATGAACGGCTTGGCGATGCGCGATGCCCTACACCGTGCGTATGTGAATGCTCGAGTGATGTCCGCCATTCCATTGAAAGGTGTGTGTGACGATTACAATTGGGCCGATGCAATCAGTCAACTGCGCCAAGGACGAGTCGTTATTTTTTCAGCGGGTACCGGTAACCCTTTCTTTACGACAGATTCTGCCGCTTGCCTACGTGGTATCGAAATTGAAGCGGATGTCGTATTGAAAGCAACAAAAGTTGACGGTGTCTACAGTGCTGACCCTGTGGCTAACCCTGATGCTCAACTGTATGATAAACTTGCTTACAGCGATGTACTGGATAAAGAACTGAAAGTGATGGA-----------------------------------------

>Vibrio_diabolicus_LMG_19805T

--------------------------------------------------------------------------------------------------------------ACAAGAAGTTAAAGAACTGGTTGAACTGGGTGTTCAGGTTGGTGTAGTTATCGGTGGCGGTAACTTGTTCCGTGGTGCAGGCCTTGCTGAAGCTGGTATGAACCGCGTAGTTGGTGACCACATGGGTATGCTTGCTACAGTAATGAATGGCCTAGCGATGCGCGATGCTCTTCACCGTGCATACGTGAATGCGCGCGTTATGTCTGCCATTCCTCTAAAAGGCGTATGTGACGACTATAATTGGGCTGATGCGATCCGTGAGCTTCGCCAAGGCCGTGTTGTCATCTTCTCTGCTGGTACGGGTAACCCGTTCTTTACAACGGATTCTGCTGCTTGCCTACGTGGTATTGAAATCGAAGCGGATGTTGTACTAAAAGCGACGAAAGTTGATGGTGTATTTACTGCTGACCCTGTAGCAAACCCAGACGCAGAGCTGTATGATAAGCTATCTTACGCAGAAGTTCTGGATAAAGAGCTGAAAGTGATGGACT---------------------------------------

>Vibrio_diazotrophicus_LMG_7893T

--------------------------------------------------------------------------------------------------------------TCAAGAAGTGAAAGAACTGGTGGAGCTTGGTGTCCAAGTGGGCGTTGTTATCGGTGGCGGTAACTTATTCCGTGGCGCTGGTCTGGCGAAAGCAGGCATGAACCGTGTTGTGGGTGACCACATGGGGATGCTAGCGACAGTGATGAATGGTTTGGCAATGCGTGATGCACTTCACCGTGCGTATGTGAATGCTCGTGTAATGTCTGCAATTCCATTGAATGGTGTGTGTGACGATTACAACTGGGCAGATGCTATCAGCCAGCTACGTGGCGGTCGTGTTGTGATTTTCTCGGCAGGTACTGGTAACCCATTCTTCACCACTGATTCAGCTGCGTGTTTGCGCGGTATTGAAATTGAAGCGGATATCGTTCTAAAAGCAACGAAAGTTGATGGAGTGTTCTCGGCTGACCCTGTAGCTAACCCAGATGCTCAGTTGTATGATAAACTTTCTTACAACGACGTTCTTGA--------------------------------------------------------------

>Vibrio_ezurae_LMG_19970T

--------------------------------------------------------------------------------------------------------------TCAGGAGATAAAAGAGTTAGTTGAACTGGGTGTCGAAGTGGGTGTTGTGATCGGCGGTGGTAACCTTTTCCGTGGCGCAGGTCTTGCAGAAGCAGGCATGAATCGCGTAGTAGGCGATCACATGGGTATGCTGGCAACCGTAATGAACGGCCTTGCAATGCGCGATGCTCTTCACCGTGCTTATGTTAACGCTCGCGTAATGTCTGCGATTCAACTGAAAGGCGTGTGTGACGACTACAACTGGGCGGATGCGATTCGCGAATTACGTCAAGGCCGTGTAGTGATCTTCTCTGCAGGTACAGGTAACCCGTTCTTCACTACAGACTCAGCAGCATGTCTACGCGGCATCGAAATCGAAGCGGATCTTGTATTAAAAGCAACAAAAGTTGACGGCGTATACAATGCTGACCCTGCAAAGAACCCAGACGCTGAACTTTACAGCCAGTTAACGTACAATGAAGTTCTAGATAAAGAGCTTAAAGTTATGGA-----------------------------------------

>Vibrio_fischeri_LMG_4414T

--------------------------------------------------------------------------------------------------------------TCAAGAAGTAAAAGAGCTGATCGAGCTTGGTGTTCAAGTTGGTGTTGTTATCGGTGGTGGTAACCTTTTCCGTGGTGCAGGTCTTGCAGAAGCAGGCATGAACCGTGTAGTAGGTGATCACATGGGTATGCTAGCGACAGTAATGAATGGCTTAGCGATGCGTGATGCACTACACCGTGCATACGTAAATGCTCGAGTAATGTCAGCTATCCCTCTTAAAGGTGTATGTGACGATTACAACTGGGCTGATGCTATCGCTCAATTACGTCAAGGCCGTGTGGTTATTTTCTCAGCTGGTACAGGTAACCCATTCTTCACAACAGATTCTGCTGCATGTCTACGTGGTATTGAAATTGAAGCTGATGTTGTTTTAAAAGCAACAAAAGTAGATGGTGTATTTACTGCTGATCCAGTATCTAACCCTGATGCAGAATTGTATGATAAACTGTCTTACAACAGCGTTCTTGAAAAAGAATTA----------------------------------------------------

>Vibrio_fluvialis_LMG_7894T

--------------------------------------------------------------------------------------------------------------TCAAGAAGTGAAAGAACTGGTAGAACTTGGTGTTCAGGTTGGCGTTGTTATCGGTGGCGGTAACTTGTTCCGTGGTGCCGGTCTGGCAGCAGCAGGTATGAACCGCGTCGTTGGTGATCACATGGGTATGTTGGCAACAGTAATGAATGGTCTGGCAATGCGTGATGCGCTGCACCGTGCTTACGTGAACGCACGTGTGATGTCGGCGATTCCTCTGAAAGGCGTGTGTGACGATTACAACTGGGCAGATGCTATCAGCCAACTGCGCCAGGGCCGCGTAGTGATTTTCTCTGCTGGTACAGGCAACCCATTCTTTACCACTGATTCAGCAGCCTGTCTGCGTGGTATCGAAATTGAAGCAGACGTAGTTCTCAAAGCGACGAAAGTAGACGGAGTTTATAGTGCAGACCCGGTAGCCAACCCAGACGCACAACTGTATGATAAGCTCGCATACAACGATGTACTTGATAAAGAATTGAAAGTGATGG------------------------------------------

>Vibrio_fortis_LMG_21557T

--------------------------------------------------------------------------------------------------------------TCAAGAAGTAAAAGAATTGGTTGAACTAGGTGTTCAAGTAGGTGTTGTAATCGGTGGTGGTAACCTTTTCCGCGGTGCAGGTCTTGCTGAAGCAGGCATGAACCGCGTTGTAGGCGACCACATGGGTATGCTAGCAACAGTAATGAACGGCCTAGCTATGCGTGATGCTCTGCACCGCGCTTACGTTAATGCACGTGTAATGTCTGCTATCCCTCTGAAGGGCGTGTGTGACGACTACAACTGGGCTGATGCGATTTCTCAACTTCGTCAAGGTCGTGTTGTTATCTTCTCTGCTGGTACAGGTAACCCGTTCTTCACTACAGATTCTGCTGCATGTCTACGCGGTATCGAGATTGAAGCTGACGTTGTTCTAAAAGCAACAAAAGTAGATGGTGTATTTACGGCTGACCCAGTAGCCAACCCAGACGCAGAGCTGTATGATACTTTGTCTTACAACACAGTTCTTGAGAAAGAGCTAAAAGTAATGGACTGGGC-----------------------------------

>Vibrio_fusnissii_LMG_7910T

--------------------------------------------------------------------------------------------------------------TCAAGAAGTGAAAGAACTGGTGGAACTGGGTGTTCAGGTGGGCGTTGTTATCGGTGGTGGTAACTTGTTCCGTGGCGCAGGCCTAGCTGCTGCTGGTATGAACCGTGTTGTGGGTGACCACATGGGGATGTTGGCAACAGTAATGAATGGCTTGGCAATGCGTGATGCACTGCACCGTGCTTACGTGAACGCACGTGTGATGTCTGCCATTCCTCTCAAAGGTGTGTGTGACGATTACAATTGGGCCGATGCGATTAGCCAACTACGCCAAGGCCGTGTGGTCATTTTCTCGGCGGGTACAGGCAACCCATTCTTTACAACCGATTCAGCAGCTTGTCTGCGCGGTATCGAAATTGAAGCGGACGTAGTTCTCAAAGCGACAAAAGTAGACGGCGTTTACAGTGCTGACCCGGTAGCCAACCCTGACGCACAACTGTATGATAAGCTCGCATACAACGATGTACTTGATAAAGAATTGAAAGTGATGGA-----------------------------------------

>Vibrio_gallicus_LMG_21330T

--------------------------------------------------------------------------------------------------------------TCAAGAAGTTAAAGAACTTGTTGAGTTGGGTGTAGAAGTGGGCGTCGTTATCGGCGGCGGCAACCTTTTCCGTGGTGCAGGTTTGGCTGAAGCAGGCATGAACCGTGTTGTTGGCGACCATATGGGAATGCTAGCGACAGTAATGAATGGCTTGGCAATGCGTGATGCACTGCATCGTGCTTATGTTAATGCCCGCGTTATGTCGGCTATCCCACTCAAAGGTGTATGTGACGACTATAACTGGGCGGATGCAATTCGTGAATTGCGTCATGGTCGTGTAGTTATTTTCTCAGCTGGAACAGGTAACCCATTCTTTACTACAGACTCAGCAGCTTGTCTGCGTGGTATCGAAATTGAAGCGGATATCGTTCTTAAAGCAACAAAAGTTGACGGCGTATACAATGCTGACCCAGCGAAGAACCCAGACGCTGAACTTTATGAGAAGTTGTCGTACAATGCTGTATTA----------------------------------------------------------------

>Vibrio_gazogenes_LMG_19540T

--------------------------------------------------------------------------------------------------------------TCAGGAAGTGAAAGAACTGGTTGAGTTAGGCGTTCAGGTCGGTGTTGTGATTGGTGGTGGCAACCTATTTCGTGGCGAAGGACTTGCCAAAGCAGGGATGAATCGTGTTGTTGGTGATCATATGGGGATGCTGGCAACCGTCATGAACGGGCTGGCGATGCGTGACGCACTGCATCGCGCTTATGTCAATGCCCGAGTCATGTCTGCTATTCCACTGAATGGTGTGTGTGATAATTATAATTGGGCCAATGCGATCAGTCAGCTTCGTCAGGGACGCGTGGTGATCTTTTCAGCAGGAACCGGTAACCCTTTCTTTACAACCGATTCTGCTGCATGTTTACGTGGTATTGAAATTGAAGCTGATGTTGTGCTCAAAGCCACAAAAGTTGATGGTGTTTATAGTGCGGATCCGGTAGCAA---------------------------------------------------------------------------------------------------------------

>Vibrio_halioticoli_LMG_18542T

--------------------------------------------------------------------------------------------------------------TCAGGAAGTAAAAGAGTTAGTTGAACTAGGTGTAGAAGTGGGCGTTGTTATCGGCGGTGGTAACCTTTTCCGTGGCGCAGGTCTTGCAGAAGCAGGTATGAATCGCGTCGTTGGCGATCACATGGGTATGCTAGCAACGGTAATGAACGGCCTTGCAATGCGTGATGCTCTGCACCGTGCTTATGTTAACGCTCGCGTAATGTCTGCTATTCAACTGAAAGGCGTGTGTGACGACTACAATTGGGCGGATGCCATTCGTGAGTTACGTCAAGGCCGCGTGGTTATTTTCTCTGCAGGTACAGGTAACCCGTTCTTCACAACAGACTCTGCTGCATGTCTACGTGGTATCGAAATTGAAACTGATCTAGTACTTAAAGCGACAAAAGTTGACGGCGTATACAATGCTGACCCTGCGAAGAACCCAGACGCTGAACTTTACAGCCAGTTAACGTACAATGAAGTTCTAGATAAAGAGCTTAAAGTTATG-------------------------------------------

>Vibrio_hepatarius_LMG_20362T

--------------------------------------------------------------------------------------------------------------ACAAGAAGTAAAAGAGTTGGTTGAACTTGGTGTTCAAGTAGGTGTGGTTATCGGTGGTGGTAACCTATTCCGCGGTGCAGGTCTAGCGGAAGCTGGTATGAACCGTGTTGTTGGTGACCACATGGGTATGCTAGCAACAGTAATGAATGGCCTAGCAATGCGTGATGCTCTTCACCGTGCATACGTAAACGCTCGCGTAATGTCAGCAATTCCTCTTAAAGGTGTGTGTGACGACTACAATTGGGCTGACGCTATCCGTGAACTTCGCCAAGGCCGTGTAGTGATTTTCTCTGCAGGTACTGGTAACCCGTTCTTTACTACAGATTCTGCTGCATGTTTGCGCGGTATTGAAATTGAAGCTGACGTAGTTCTAAAAGCGACAAAAGTAGATGGTGTATTTACGGCTGACCCTGTAGCAAACCCTGACGCAGAGCTGTATGATAAGCTTTCATTCAGTGCAGTTCTTG---------------------------------------------------------------

>Vibrio_hispanicus_LMG_13240T

--------------------------------------------------------------------------------------------------------------TCAAGAAGTGAAAGAACTGGTTGAACTTGGCGTTCAAGTAGGGGTTGTTATCGGTGGTGGTAACTTATTCCGTGGTGCTGGTTTAGCAGCCGCTGGCATGAACCGTGTTGTTGGTGACCACATGGGCATGCTTGCAACGGTAATGAACGGCCTTGCGATGCGTGATGCTCTACACCGCGCTTACGTAAATGCGCGCGTAATGTCAGCCATTCCTCTAAACGGCGTATGTGATGACTATAACTGGGCGGATGCGATTAGCCAATTGCGCCAAGGTCGCGTAGTGATCTTCTCTGCTGGTACGGGCAACCCATTTTTTACGACTGATTCAGCTGCATGTTTACGTGGCATCGAAATTGAAGCCGATATCGTACTAAAAGCAACCAAAGTTGACGGTGTATTCACAGCTGACCCAGTAGCGAACCCAGAAGCTGAGTTGTATAATAAGCTTTCAT------------------------------------------------------------------------------

>Vibrio_ichthyoenteri_LMG_19664T

--------------------------------------------------------------------------------------------------------------TCAAGAAGTAAAAGAGTTAGTTGAACTGGGCGTTCAAGTTGGTGTCGTTATCGGTGGTGGTAACTTATTCCGTGGTGCAGGCCTTGCTGAAGCGGGCATGAACCGTGTTGTTGGCGACCATATGGGTATGCTAGCAACGGTAATGAATGGCCTAGCATTGCGTGATGCACTGCACCGTGCATACGTGAATGCGCGTGTAATGTCGGCAATTCCACTGAAAGGTGTTTGTGACGATTACAATTGGGCTGATGCGATTCGTGAATTACGTCAAGGCCGTGTTGTTATCTTCTCTGCAGGTACTGGTAATCCATTCTTCACAACCGATTCAGCAGCGTGTCTACGTGGCATTGAAATCGAAGCTGATGTAGTTCTAAAAGCAACCAAAGTAGATGGCGTATTTACCTCTGACCCTGTGGCAAACCCAGACGCAGAGCTGTATGATAAGTTGTCTTACGCAGAGATTCTTGACAAAGAACTTAAAGTGATG-------------------------------------------

>Vibrio_kanaloaei_LMG_20539T

--------------------------------------------------------------------------------------------------------------TCAAGAAGTAAAAGAATTGGTTGAACTAGGTGTTCAAGTCGGTGTTGTTATCGGTGGCGGTAACCTTTTCCGCGGTGCTGGTCTTGCTGAAGCCGGTATGAATCGCGTTGTTGGTGACCACATGGGTATGCTTGCAACGGTAATGAACGGCCTTGCAATGCGTGACGCTCTACACCGTGCTTATGTGAATGCACGTGTAATGTCAGCAATCCCTCTTAAAGGTGTGTGTGACGACTACAATTGGGCAGACGCAATCAGTCAACTACGTCAAGGTCGTGTTGTGATCTTCTCTGCTGGTACTGGTAACCCATTCTTTACTACGGATTCTGCTGCATGTTTACGTGGTATCGAAATCGAAGCTGACGTCGTTCTAAAAGCGACAAAGGTAGATGGTGTATTTACTGCTGACCCTGTAGCAAACCCAGAAGCAGAGCTGTATGATACGTTGTCT-------------------------------------------------------------------------------

>Vibrio_lentus_LMG_21034T

--------------------------------------------------------------------------------------------------------------TCAAGAAGTAAAAGAATTGGTTGAACTAGGTGTTCAAGTAGGTGTTGTTATCGGTGGCGGTAACCTTTTCCGTGGCGCAGGCCTTGCAGAAGCAGGTATGAACCGCGTTGTTGGTGACCACATGGGTATGCTTGCAACGGTAATGAACGGCCTTGCAATGCGTGACGCTCTACACCGTGCTTACGTAAACGCACGTGTAATGTCTGCAATCCCTCTTAAAGGTGTGTGTGACGATTACAACTGGGCAGATGCAATCAGCCAACTACGTCAAGGTCGCGTTGTGATCTTCTCTGCAGGTACTGGTAACCCATTCTTCACTACAGATTCTGCTGCGTGTCTACGTGGTATCGAAATCGAAGCTGACGTAGTTCTAAAAGCGACGAAAGTAGATGGTGTATTTACTGCTGACCCAGTAGCAAACCCAGACGCAGAGCTGTATGATACTTTGTCTTACAACA------------------------------------------------------------------------

>Vibrio_marinus_R-40493T

--------------------------------------------------------------------------------------------------------------TCAAGAAGTAAAAGAGCTAGTAGAACTGGGTGTTCAAGTTGGTGTTGTAATTGGTGGTGGTAACCTATTCCGTGGTGCGGGTCTTGCTGAAGCTGGCATGAACCGTGTTGTTGGTGACCACATGGGTATGCTTGCGACTGTAATGAATGGTCTAGCAATGCGTGATGCTCTACACCGTGCTTATGTTAATGCTCGTGTAATGTCAGCTATCCCACTTAAAGGTGTATGTGACGATTACAACTGGGCTGATGCGATTCGCGAACTTCGTCAAGGTCGCGTAGTTATCTTCTCAGCAGGTACTGGTAACCCATTCTTCACAACAGACTCTGCTGCGTGTCTACGTGGTATTGAAATCGAAGCTGATGTAGTTCTTAAAGCAACCAAAGTAGATGGCGTATTTACTGCTGATCCAGTGGCTAACCCAGATGCTGTATTATGTGATAAGCTTTCATACAACTCAGTTTTAGATAAAGAACTGAAAGTAATGGA-----------------------------------------

>Vibrio_mimicus_LMG_7896T

--------------------------------------------------------------------------------------------------------------TCAGGAAGTAAAAGAACTGGTTGAACTGGGCGTACAAGTGGGTGTTGTGATTGGTGGGGGTAACCTGTTCCGTGGTGCAGGCCTTGCAAAAGCAGGCATGAACCGCGTTGTGGGTGACCATATGGGTATGCTGGCAACCGTGATGAACGGATTGGCGATGCGTGATGCATTGCACCGTGCTTATGTTAACGCACGTCTAATGTCTGCTATTCCTCTGAATGGTGTGTGTGACGATTACAGCTGGTCCGATGCGATTCGTGAATTGCGCCAAGGCCGTGTGGTAATTTTCGCTGCGGGTACTGGTAATCCTTTCTTTACTACCGATTCTGCAGCGTGTCTGCGCGGTATTGAGATTGAAGCTGACGTGGTTCTCAAAGCGACGAAAGTAGATGGTGTTTACAGCGCTGACCCGGTAGCCAACCCAGATGCACAACTGTATGATAAGCTTGCTTACAACGATGTGCTT----------------------------------------------------------------

>Vibrio_mytili_LMG_19157T

--------------------------------------------------------------------------------------------------------------GCAAGAAGTGAAAGAACTGGTTGAACTGGGTGTTCAGGTTGGTGTAGTTATCGGTGGTGGTAACTTGTTCCGTGGTGCAGGCCTTGCAGAAGCAGGTATGAACCGTGTCGTGGGTGACCATATGGGCATGCTTGCTACGGTTATGAATGGCCTAGCTATGCGTGATGCGCTTCACCGTGCTTATGTTAATGCTCGTGTTATGTCTGCCATTCCGCTAAAAGGCGTGTGTGACGACTACAATTGGGCGGATGCGATTCGTGAGCTACGTCAAGGCCGCGTAGTTATCTTCTCTGCGGGTACTGGTAACCCATTCTTTACCACGGACTCTGCGGCATGTCTGCGTGGTATCGAAATTGAAGCAGATATCGTGCTAAAAGCAACGAAAGTTGATGGTGTATTTACTGCTGACCCAGTAGCAAACCCAGACGCAGAACTGTATGATAAGCTATCTTACGCTGAAGTTCTGGATAAAGAGCT-----------------------------------------------------

>Vibrio_natriegens_LMG_10935T

--------------------------------------------------------------------------------------------------------------GCAAGAAGTTAAAGAACTTGTTGAACTAGGTGTTCAGGTTGGTGTAGTTATCGGTGGCGGTAACCTGTTCCGTGGTGCTGGTCTTGCAGAAGCTGGCATGAACCGTGTAGTGGGTGACCACATGGGTATGCTTGCTACAGTAATGAACGGCCTTGCAATGCGTGACGCTCTGCACCGTGCATACGTAAACGCGCGTGTAATGTCTGCAATTCCTCTTAAAGGCGTATGTGACGACTATAATTGGGCTGATGCGATTCGTGAACTTCGCCAAGGCCGCGTTGTTATCTTCTCTGCAGGTACAGGTAACCCATTCTTTACAACGGATTCAGCAGCGTGTCTACGTGGTATCGAAATCGAAGCAGACGTAGTTCTAAAAGCGACAAAAGTTGATGGTGTATTTACTGCTGACCCAGTAGCAAACCCAGACGCAGAGCTGTATGATAAGCTATCTTACGCAGAAGTTCTGGATAAAGAGCTTAAAGTAATG-------------------------------------------

>Vibrio_navarrensis_LMG_15976T

--------------------------------------------------------------------------------------------------------------GCAGGAAGTCAAAGAACTGGTTGAACTGGGTGTGCAAGTCGGCGTGGTGATCGGTGGCGGCAACCTATTCCGTGGTGCTGGCCTTGCTGAAGCGGGCATGAACCGCGTTGTGGGCGATCACATGGGTATGCTGGCAACGGTCATGAATGGCCTAGCGATGCGTGATGCACTGCACCGTGCGTACGTAAATGCACGAGTGATGTCTGCCATCCCACTGAAAGGGGTATGTGATGACTATAATTGGGCAGACGCGATTCGCGAACTGCGTCAAGGCCGCGTGGTGATTTTCTCTGCGGGTACGGGCAACCCATTCTTCACGACCGACTCTGCCGCGTGTTTGCGTGGTATCGAAATTGAGGCCGATGTGGTTCTCAAAGCAACGAAAGTGGATGGCGTATTCAGCGCAGACCCGGTAGCCAATCCAGACGCAGAGCTGTATGATAAACTGGCTTAC----------------------------------------------------------------------------

>Vibrio_neonatus_LMG_19972

--------------------------------------------------------------------------------------------------------------TCAGGAAATAAAAGAGTTAGTTGAACTTGGCGTAGAAGTAGGTGTTGTTATCGGTGGTGGTAACCTTTTCCGTGGCGCAGGTCTTGCAGAAGCTGGCATGAATCGCGTAGTAGGCGATCACATGGGTATGCTAGCAACGGTAATGAACGGCCTTGCAATGCGTGATGCTCTGCACCGTGCTTATGTTAATGCTCGCGTAATGTCTGCGATTCAACTGAAAGGCGTGTGTGACGATTACAACTGGGCAGATGCTATTCGTGAATTACGTCACGGCCGTGTGGTTATTTTCTCTGCAGGTACAGGTAACCCATTCTTCACAACAGATTCTGCGGCATGTCTACGTGGTATCGAAATCGAAGCAGATCTAGTGCTAAAAGCAACAAAAGTTGACGGCGTATACAATGCTGACCCAGCGAAGAACCCAGACGCTGAACTTTACAGCCAGTTAACGTACAATGAAGTTC------------------------------------------------------------------

>Vibrio_neptunius_LMG_20536T

--------------------------------------------------------------------------------------------------------------TCAAGAAGTAAAAGAACTGGTTGAACTGGGTGTTCAGGTCGGTGTGGTTATCGGGGGTGGTAACTTGTTCCGTGGTGCAGGGCTTGCTGAAGCGGGTATGAACCGCGTTGTCGGTGACCACATGGGTATGTTAGCAACAGTAATGAACGGTTTGGCGATGCGTGATGCTTTGCACCGTGCCTACGTCAATGCTCGTGTTATGTCTGCAATCCCACTAAAAGGTGTGTGTGACGATTACAACTGGGCAGATGCAATCCGCGAACTGCGCCAAGGTCGTGTTGTTATCTTCTCAGCAGGTACAGGTAACCCATTCTTCACAACCGATTCTGCTGCATGTCTACGTGGAATTGAAATTGAAGCTGACGTAGTTCTAAAAGCGACAAAAGTTGATGGTGTATTTACGGCTGACCCAGTAGCCAACCCTGACGCAGAGCTGTATGATAACCTGAGCTACGCAGAAGTTCTTGATAAAGAACTAAAAGTGATG-------------------------------------------

>Vibrio_nereis_LMG_3895T

--------------------------------------------------------------------------------------------------------------TCAAGAGGTTAAAGAACTGGTTGAACTAGGCGTTCAGGTTGGTGTAGTTATCGGTGGTGGTAACTTATTCCGTGGTGCAGGTCTGGCTGAAGCTGGTATGAACCGCGTTGTTGGCGACCACATGGGTATGCTGGCGACTGTGATGAACGGTCTTGCGATGCGTGATGCACTGCACCGTGCTTACGTTAACGCTCGCGTGATGTCTGCGATTCCTCTAAAAGGTGTATGTGACGATTACAACTGGGCAGACGCAATCCGTGAACTTCGTCAAGGTCGTGTGGTGATCTTCTCGGCTGGTACAGGTAACCCGTTCTTCACAACAGACTCAGCGGCATGTCTACGTGGTATCGAAATCGAAGCGGATGTTGTACTAAAAGCAACAAAAGTGGATGGCGTATTTACAGCTGACCCAGTAGCAAACCCAGACGCAGAGCTGTATGATAAGCTTTCTTACGCTGAAGTTCTTGATAAAGAACTGAAAGTGATGGACG---------------------------------------

>Vibrio_ordalii_LMG_13544T

--------------------------------------------------------------------------------------------------------------ACAGGAAGTAAAAGAACTGGTTGAACTGGGTGTTCAAGTGGGTGTTGTCATCGGCGGCGGTAACTTATTCCGTGGCGCAGGTCTTGCTGAAGCGGGCATGAACCGTGTTGTGGGCGACCACATGGGAATGCTGGCAACCGTAATGAATGGTTTGGCAATGCGTGATGCTTTGCACCGTGCTTATGTGAATGCGCGCGTGATGTCTGCCATTCCTCTTAAAGGTGTGTGTGACGATTACAATTGGGCCGATGCTATCAGCCAACTACGTCAAGGCCGTGTTGTGATTTTCTCTGCGGGTACTGGTAACCCATTCTTCACGACCGATTCTGCTGCCTGTCTACGCGGTATTGAAATTGAAGCCGATGTGGTTCTAAAAGCGACAAAAGTAGATGGCGTATTTAGTGCAGACCCAGTGGCAAACCCAGACGCAGAACTGTATGATAAGCTGTCTTATACAACCGTGCTTGAAAAAGAGCTAAAAGTGATGGA-----------------------------------------

>Vibrio_orientalis_LMG_7897T

--------------------------------------------------------------------------------------------------------------ACAAGAAGTAAAAGAGTTGGTTGAACTTGGTGTTCAAGTTGGTGTGGTTATCGGTGGTGGTAACCTATTCCGCGGTGCAGGCCTAGCCGAAGCGGGTATGAACCGTGTTGTTGGTGACCACATGGGTATGCTAGCAACAGTAATGAATGGCCTAGCCATGCGTGATGCTCTTCACCGTGCATACGTGAACGCTCGCGTAATGTCAGCAATTCCTCTTAAAGGTGTGTGTGACGATTACAACTGGGCTGACGCTATCCGCGAACTTCGCCAAGGCCGTGTAGTGATTTTCTCTGCAGGTACTGGTAACCCGTTCTTTACTACAGATTCTGCTGCATGTTTGCGCGGTATTGAAATTGAAGCTGACGTAGTTCTAAAAGCGACAAAAGTAGATGGCGTATTTAGCGCTGACCCTGTAGCAAACCCTGACGCAGAGCTGTATGATAAGCTTTCATTCAATGCAGTTCTTG---------------------------------------------------------------

>Vibrio_pacinii_LMG_19999T

--------------------------------------------------------------------------------------------------------------CCAAGAAATAAAAGAACTGGTTGAACTGGGTGTTCAGGTCGGTGTTGTTATCGGTGGCGGTAACTTGTTCCGTGGTGCAGGTCTGGCAGAAGCGGGTATGAACCGCGTTGTTGGTGACCACATGGGGATGCTGGCGACAGTAATGAATGGCCTAGCGATGCGCGATACACTGCACCGTGCTTATGTCAATGCGCGAGTAATGTCTGCCATCCCATTAAAAGGTGTGTGTGACGATTACAATTGGGCCGATGCGATTCGTGAACTTCGTCAAGGCCGCGTGGTGATCTTCTCTGCAGGTACTGGTAACCCATTTTTCACGACCGATTCTGCTGCTTGTTTGCGCGGTATCGAAATTGAAGCTGACGTAGTTCTAAAAGCGACAAAAGTAGATGGCGTATTTAGCGCAGACCCTGTAGCAAACCCAGACGCAGAACTGTATGATAAGCTTTCATACGAAACAGTGCTTGAAAAAGAACTGAAAGTAATG-------------------------------------------

>Vibrio_parahaemolyticus_LMG_2850T

--------------------------------------------------------------------------------------------------------------ACAAGAAGTTAAAGAACTTGTTGAACTGGGTGTTCAGGTTGGTGTAGTTATCGGTGGCGGTAACCTGTTCCGTGGCGCTGGTCTTGCAGAAGCTGGTATGAACCGTGTAGTGGGTGACCACATGGGTATGCTTGCTACAGTAATGAACGGCCTTGCGATGCGTGACGCTCTTCACCGTGCATACGTAAATGCGCGCGTAATGTCTGCAATTCCTCTTAAAGGCGTATGTGACGACTACAATTGGGCTGATGCAATTCGCGAACTTCGTCAAGGCCGCGTTGTAATCTTCTCAGCAGGTACTGGTAACCCATTCTTCACAACAGACTCTGCTGCGTGTCTACGTGGTATCGAAATCGAAGCTGACGTAGTTCTAAAAGCAACGAAAGTTGATGGCGTATTTACTGCTGACCCAGTAGCAAACCCAGACGCAGAGCTGTATGATAAGCTATCTTACGCAGAAGTTCTGGATAAAGAGCTTAAAGTAATGG------------------------------------------

>Vibrio_pectenicida_LMG_19642T

--------------------------------------------------------------------------------------------------------------TCAAGAAGTAAAAGAGCTGGTGGAACTGGGTGTACAAGTTGGTGTGGTCATCGGAGGGGGGAACCTTTTCCGAGGTGCGGGACTTGCAGAAGCTGGCATGAACCGAGTTGTTGGTGATCACATGGGTATGTTGGCCACGGTGATGAATGGTCTTGCGATGCGTGATGCTTTGCATCGTGCTTATGTAAATGCGCGTGTTATGTCTGCTATTCCTCTAAAAGGTGTATGTGATGATTACAACTGGGCAGATGCTATTCGTGAACTGCGCCAGGGAAGAGTGGTAATTTTCTCGGCAGGTACGGGGAATCCATTTTTTACTACTGATTCTGCAGCCTGTTTACGTGGCATTGAAATTGAAGCAGATGTGGTTTTAAAAGCGACAAAAGTAGATGGTGTATTTACCGCTGATCCAGTAGCTAATCCAGATGCAGAGTTGTATGAT----------------------------------------------------------------------------------------

>Vibrio_pelagius_LMG_3897T

--------------------------------------------------------------------------------------------------------------TCAAGAAGTAAAAGAATTGGTTGAACTAGGTGTTCAAGTAGGTGTTGTAATTGGTGGCGGTAACCTTTTCCGTGGCGCAGGTCTTGCTGAAGCTGGTATGAACCGCGTTGTGGGTGACCACATGGGTATGCTTGCAACAGTAATGAACGGCCTTGCTATGCGTGACGCTCTACACCGTGCTTACGTAAATGCACGTGTAATGTCTGCAATCCCTCTTAAAGGTGTATGTGACGATTACAACTGGGCTGATGCGATTTCTCAACTTCGTCAGGGTCGTGTTGTGATCTTCTCTGCGGGTACAGGTAACCCATTCTTCACTACAGATTCTGCTGCATGTCTACGTGGTATCGAGATTGAAGCTGATGTTGTGTTAAAAGCAACTAAAGTAGATGGTGTATTTACGGCTGACCCAGTAGCAAACCCAGACGCAGAGCTGTATGATACTTTGTCTTACAACACAGTTCTTGAGAAAGAGCTAAAAGTAATGG------------------------------------------

>Vibrio_penaeicida_LMG_19663T

--------------------------------------------------------------------------------------------------------------TCAAGAAGTAAAAGAACTGGTTGAACTGGGTGTTCAAGTGGGTGTGGTTATCGGTGGTGGTAACTTGTTCCGTGGTGCTGGTTTGGCTGAAGCGGGCATGAACCGCGTTGTCGGCGACCATATGGGTATGCTAGCGACGGTAATGAATGGTCTTGCTTTACGCGATGCCCTTCACCGTGCTTACGTCAATGCGCGTGTAATGTCGGCGATTCCATTGAAAGGTGTGTGCGACGATTACAACTGGGCTGATGCAATTCGCGAGCTTCGCCAAGGTCGCGTAGTGATCTTCTCGGCAGGTACAGGTAACCCATTCTTTACTACAGATTCTGCTGCATGTTTGCGCGGTATTGAGATTGAAGCGGATGTTGTTCTTAAAGCGACAAAAGTTGATGGCGTATTTACTGCGGATCCAGTAGCAAACCCTGAAGCAGAGCTGTATGATAACCTATCGTTTAGTGAAGTTCTTGATAAAGAACTGAAAGTATG--------------------------------------------

>Vibrio_pomeroyi_LMG_20537T

--------------------------------------------------------------------------------------------------------------TCAAGAAGTAAAAGAATTGGTTGAACTAGGTGTTCAAGTAGGTGTTGTTATCGGTGGCGGTAACCTTTTCCGTGGTGCAGGTCTTGCAGAAGCTGGCATGAACCGCGTTGTTGGTGACCACATGGGTATGCTTGCAACGGTAATGAACGGCCTTGCAATGCGTGACGCTCTACACCGTGCTTACGTAAACGCACGTGTAATGTCAGCAATCCCTCTTAAAGGTGTGTGTGACGACTACAATTGGGCAGATGCAATCAGCCAACTACGTCAAGGTCGCGTTGTGATCTTCTCTGCTGGTACTGGTAACCCATTCTTCACTACGGATTCTGCTGCGTGTCTACGTGGTATCGAAATCGAAGCTGACGTAGTTCTAAAAGCGACAAAGGTAGATGGTGTATTTACTTCTGACCCTGTAGCAAACCCAGACGCAGAGCTGTATGATACGTTGTCTTACAACACGGTTCTTGATAAAGAACTTAAAGTAATGG------------------------------------------

>Vibrio_proteolyticus_LMG_3772T

--------------------------------------------------------------------------------------------------------------GCAAGAGGTCAAAGAACTGGTTGAACTGGGTGTTCAGGTGGGCGTGGTTATCGGTGGTGGTAACCTGTTCCGCGGTGCAGGTCTGGCGGAAGCGGGTATGAACCGTGTTGTGGGTGACCACATGGGGATGCTGGCAACGGTAATGAACGGCTTGGCGATGCGTGATGCCTTGCACCGTGCTTACGTGAACGCACGTGTAATGTCGGCAATTCCACTGCAAGGTGTGTGTGACGACTACAATTGGGCAGATGCCATCCGTGAACTGCGTCAGGGCCGCGTGGTGATTTTCTCGGCAGGTACCGGCAACCCATTCTTTACAACCGATTCTGCGGCGTGTCTGCGTGGTATTGAAATTGAAGCCGATGTGGTGCTTAAAGCGACCAAAGTCGATGGCGTATTTAGCGCAGACCCGGTAGCAAACCCGGACGCAGAACTGTATGATAAGTTGTCTTACAACGCTGTTCTGGAAAAAGAACTGAAAGTGATGGAC----------------------------------------

>Vibrio_rumoiensis_LMG_20038T

--------------------------------------------------------------------------------------------------------------TCAAGAAATTAAAGAACTCGTTGAACTTGGTGTACAAGTTGGCGTTGTGATTGGTGGTGGTAACTTATTCCGTGGCGCTGGTTTAGCCGAAGCGGGGATGAACCGTGTGGTTGGTGATCATATGGGAATGTTAGCAACCGTCATGAATGGACTCGCAATGCGTGATGCGCTTCATCGTGCTTATGTGAATGCGCGTGTGATGTCAGCTATTCAATTAAAAGGTGTGTGTGACGATTACAATTGGGCTGATGCGATTAGCCAACTTCGTCAAGGCCGAGTGGTTATCTTCTCTGCAGGTACTGGTAACCCATTCTTTACGACTGATTCAGCAGCATGCTTGCGTGGTATCGAAATTGAAGCAGATATCGTTCTAAAAGCGACCAAAGTGGATGGTGTTTATACAGCTGATCCGGTTGCTAACCCAGATGCAGTTTTGTGTGATAAACTCAGCTAC----------------------------------------------------------------------------

>Vibrio_splendidus_LMG19031T

--------------------------------------------------------------------------------------------------------------TCAAGAAGTAAAAGAATTGGTTGAACTAGGTGTTCAAGTAGGCGTTGTTATCGGTGGCGGTAACCTTTTCCGTGGTGCAGGCCTTGCTGAAGCAGGTATGAACCGCGTTGTTGGTGACCACATGGGTATGCTAGCAACGGTAATGAACGGCCTTGCAATGCGTGACGCCCTGCACCGTGCTTACGTAAATGCACGTGTAATGTCAGCTATCCCTCTTAAAGGTGTGTGTGACGACTACAACTGGGCAGATGCAATCAGCCAACTACGTCAAGGTCGTGTTGTGATCTTCTCAGCTGGTACTGGTAACCCATTCTTCACTACAGATTCTGCTGCGTGTCTACGTGGTATCGAAATTGAAGCTGACGTAGTTCTAAAAGCGACAAAGGTAGATGGCGTATTTACTGCTGACCCAGTAGCAAACCCAGACGCAGAGCTGTATGATAC--------------------------------------------------------------------------------------

>Vibrio_superstes_LMG_21323T

--------------------------------------------------------------------------------------------------------------TCAAGAAGTAAAAGAGTTGGTTGAGCTAGGTGTAGAAGTAGGTGTGGTTATCGGCGGTGGCAACTTGTTCCGCGGTGCTGGTCTTGCAGAAGCTGGCATGAACCGCGTTGTAGGCGACCATATGGGTATGCTAGCAACAGTGATGAATGGCCTAGCAATGCGTGATGCATTGCACCGTGCATACGTGAATGCTCGCGTAATGTCAGCGATTCAACTAAAAGGTGTGTGTGACGATTACAACTGGGCTGACGCTATCCGCGAACTTCGTCAAGGTCGCGTAGTGATCTTCTCTGCAGGAACGGGTAACCCATTCTTCACAACGGACTCTGCTGCTTGTCTTCGTGGTATCGAGATCGAAGCGGATCTAGTACTAAAAGCAACAAAAGTTGACGGCGTATACAATGCTGACCCTGCAACGAACCCAGACGCTGAACTTTACAGCAAGTTATCGTACAATGAA----------------------------------------------------------------------

>Vibrio_tasmanienis_LMG_20012T

--------------------------------------------------------------------------------------------------------------TCAAGAAGTAAAAGAATTAGTTGAACTAGGCGTTCAAGTTGGTGTTGTTATCGGTGGTGGTAACCTATTCCGTGGCGCAGGCCTAGCAGAAGCGGGTATGAACCGTGTTGTTGGTGACCACATGGGTATGCTTGCAACGGTAATGAACGGCCTTGCAATGCGTGACGCTCTACACCGTGCTTACGTAAATGCACGTGTAATGTCAGCAATTCCTCTTAAAGGTGTGTGTGACGACTACAACTGGGCAGACGCAATCAGCCAACTACGTCAAGGTCGTGTTGTGATCTTCTCTGCTGGTACGGGTAACCCATTCTTTACTACAGACTCTGCTGCATGTTTACGCGGTATCGAAATCGAAGCTGACGTAGTTCTAAAAGCAACAAAAGTAGATGGTGTATTTACTGCTGACCCAGTAGCAAACCCAGACGCAGAGTTGTATGATACGTTGTCTTACAACACAAT--------------------------------------------------------------------

>Vibrio_tubiashii_LMG_10936T

--------------------------------------------------------------------------------------------------------------CCAAGAAGTAAAAGAGTTGGTTGAGCTTGGTGTTCAAGTTGGTGTTGTTATCGGTGGTGGTAACTTGTTCCGTGGTGCTGGCCTAGCTGAAGCGGGCATGAACCGTGTTGTTGGTGACCACATGGGTATGCTAGCGACGGTAATGAACGGTCTTGCGATGCGTGATGCGTTGCACCGTGCTTACGTAAACGCTCGTGTAATGTCAGCAATCCCTCTAAAAGGTGTCTGTGACGACTACAACTGGGCAGATGCGATTCGTGAACTGCGTCAAGGCCGCGTTGTTATCTTCTCTGCAGGTACTGGTAACCCATTCTTTACCACAGATTCTGCTGCATGTTTGCGCGGAATCGAGATCGAAGCGGACGTAGTTCTAAAAGCGACAAAAGTAGATGGCGTATTTACTGCTGACCCAGTAGCAAACCCAGACGCAGAGCTGTATGATAAGCTTTCGTTCAATGCTGTTC------------------------------------------------------------------

>Vibrio_variabilis R-40492T

------------------------------------------------------------------------------------------------------------------------------------------------------------------------GGTAACCTATTCCGTGGTGCGGGTCTTGCTGAAGCTGGCATGAACCGTGTTGTTGGTGACCACATGGGTATGCTTGCGACTGTAATGAATGGTCTAGCAATGCGTGATGCTCTACACCGTGCTTATGTTAATGCTCGTGTAATGTCAGCTATCCCACTTAAAGGTGTATGTGACGATTACAACTGGGCAGATGCGATTCGCGAACTTCGTCAAGGTCGCGTAGTGATCTTCTCTGCTGGTACCGGTAACCCATTCTTCACAACTGACTCTGCTGCGTGTCTACGTGGTATTGAAATCGAAGCTGATGTTGTTCTTAAAGCCACCAAGGTAGATGGTGTATTCACTGCTGATCCAGTGGCTAACCCAGATGCTGTATTATGTGATAAGCTTTCGTACAACTCAGTTCTAGAAAAAGAACTGAAAGTAATGGA-----------------------------------------

>Vibrio_vulnificus_LMG_13545T

--------------------------------------------------------------------------------------------------------------TCAGGAAGTAAAAGAATTAGTAGAACTTGGTGTACAAGTCGGTGTGGTTATCGGTGGCGGTAACTTGTTCCGTGGCGCTGGTCTAGCGCAAGCAGGCATGAACCGTGTTGTGGGTGACCACATGGGGATGCTAGCAACGGTGATGAACGGCCTAGCGATGCGCGATGCGCTACACCGTGCTTACGTTAACGCTCGTGTGATGTCAGCTATCCCTTTAAAGGGAGTGTGCGACGACTACAATTGGGCAGACGCCATTCGTGAACTTCGTCAAAGTCGAGTGGTTATCTTTGCTGCAGGTACTGGCAACCCATTCTTCACTACGGATTCTGCTGCTTGCCTACGTGGTATTGAGATTGAAGCGGATGTCGTTTTAAAAGCGACTAAAGTGGATGGCGTATTTACTGCTGACCCGGTAGCCAACCCAGACGCAGAGCTGTATGATAAGCTTTCTTATACCGATGTTCTTGAAAAAGAACTGAAAGTGATGGAC----------------------------------------

>Vibrio_xuii_LMG_21346T

--------------------------------------------------------------------------------------------------------------ACAAGAAGTGAAAGAGCTGGTTGAACTAGGCGTTCAGGTTGGTGTAGTTATCGGTGGTGGTAACCTGTTCCGCGGTGCTGGTCTGGCTGAAGCTGGTATGAACCGTGTAGTTGGTGACCACATGGGTATGTTGGCTACAGTGATGAACGGTCTTGCAATGCGTGACGCTCTGCACCGTGCATACGTAAACGCTCGTGTAATGTCTGCAATTCCTCTAAAAGGTGTGTGTGACGATTACAATTGGGCAGACGCAATCCGCGAACTTCGCCAAGGCCGTGTAGTGATCTTCTCTGCGGGTACGGGTAACCCATTCTTCACGACGGATTCAGCTGCGTGTCTACGCGGTATCGAAATCGAAGCAGACGTCGTTCTTAAAGCCACTAAAGTAGATGGCGTATTTACGGCAGACCCAGTAGCAAACCCAGACGCAGAGCTGTATGATAAGCTGACTTTCGCTGAAGTTCTTGATAAAGAGCTAAAAGTGATGG------------------------------------------

>Vibrio coralliilyticus strain LMG 20984

---------------CCAGCGTATCAACGTATTCTGTTAAAACTGAGTGGTGAAGCACTTCAAGGCGAAGAAGGTTTTGGTATTGATCCTGCGATCTTGGATCGTATGGCTCAAGAAGTAAAAGAACTGGTTGAACTGGGTGTTCAGGTCGGTGTGGTTATCGGTGGTGGTAACTTGTTCCGTGGTGCAGGTCTTGCTGAAGCGGGTATGAACCGTGTTGTTGGTGACCACATGGGTATGTTAGCAACAGTAATGAATGGTTTGGCGATGCGTGATGCTTTGCACCGTGCATACGTCAATGCTCGTGTTATGTCTGCAATCCCACTAAAAGGTGTGTGTGACGATTACAACTGGGCAGACGCAATCCGTGAGTTGCGCCAAGGTCGTGTTGTTATCTTCTCAGCAGGTACAGGTAACCCATTCTTCACAACAGATTCTGCTGCATGTCTACGCGGAATTGAAATTGAAGCTGACGTAGTTCTAAAAGCGACAAAAGTTGATGGCGTATTTACGGCTGACCCGGTAGCCAACCCTGACGCAGAGCTGTATGATAACCTGAGCTACGCAGAAGTTCTTGATAAAGAACTAAAAGTGATGG------------------------------------------

>Vibrio coralliilyticus ATCC BAA-450

---------------CCAGCGTATCAACGTATTCTGTTAAAACTGAGTGGTGAAGCACTTCAAGGCGAAGAAGGTTTTGGTATTGATCCTGCGATCTTGGATCGTATGGCTCAAGAAGTAAAAGAACTGGTTGAACTGGGTGTTCAGGTCGGTGTGGTTATCGGTGGTGGTAACTTGTTCCGTGGTGCAGGTCTTGCTGAAGCGGGTATGAACCGTGTTGTTGGTGACCACATGGGTATGTTAGCAACAGTAATGAATGGTTTGGCGATGCGTGATGCTTTGCACCGTGCATACGTCAATGCTCGTGTTATGTCTGCAATCCCACTAAAAGGTGTGTGTGACGATTACAACTGGGCAGACGCAATCCGTGAGTTGCGCCAAGGTCGTGTTGTTATCTTCTCAGCAGGTACAGGTAACCCATTCTTCACAACAGATTCTGCTGCATGTCTACGCGGAATTGAAATTGAAGCTGACGTAGTTCTAAAAGCGACAAAAGTTGATGGCGTATTTACGGCTGACCCGGTAGCCAACCCTGACGCAGAGCTGTATGATAACCTGAGCTACGCAGAAGTTCTTGATAAAGAACTAAAAGTGATGG------------------------------------------

>Vibrio_coralliilyticus_LMG_20984T

--------------------------------------------------------------------------------------------------------------TCAAGAAGTAAAAGAACTGGTTGAACTGGGTGTTCAGGTCGGTGTGGTTATCGGTGGTGGTAACTTGTTCCGTGGTGCAGGTCTTGCTGAAGCGGGTATGAACCGTGTTGTTGGTGACCACATGGGTATGTTAGCAACAGTAATGAATGGTTTGGCGATGCGTGATGCTTTGCACCGTGCATACGTCAATGCTCGTGTTATGTCTGCAATCCCACTAAAAGGTGTGTGTGACGATTACAACTGGGCAGACGCAATCCGTGAGTTGCGCCAAGGTCGTGTTGTTATCTTCTCAGCAGGTACAGGTAACCCATTCTTCACAACAGATTCTGCTGCATGTCTACGCGGAATTGAAATTGAAGCTGACGTAGTTCTAAAAGCGACAAAAGTTGATGGCGTATTTACGGCTGACCCGGTAGCCAACCCTGACGCAGAGCTGTATGATAACCTGAGCTACGCAGAAGTTCTTGATAAAGAACTAAAAGTGATGG------------------------------------------

>38A

-----------------------------------------------------------------------------------------------------------------AGAAGTAAAAGAACTGGTTGAACTGGGTGTTCAGGTCGGTGTGGTTATCGGTGGTGGTAACTTGTTCCGTGGTGCAGGTCTTGCTGAAGCGGGTATGAACCGTGTTGTTGGTGACCACATGGGTATGTTAGCAACAGTAATGAACGGTTTGGCGATGCGTGATGCTTTGCACCGTGCATACGTCAATGCTCGTGTTATGTCTGCAATCCCACTGAAAGGTGTGTGTGACGATTACAACTGGGCAGATGCAATCCGCGAACTGCGCCAAGGCCGTGTTGTTATCTTCTCAGCAGGTACAGGTAACCCATTCTTCACAACCGATTCTGCTGCATGTCTACGCGGAATTGAAATTGAAGCTGACGTAGTTCTAAAAGCGACAAAAGTTGATGGTGTATTTACGGCTGACCCAGTAGCCAACCCTGACGCAGAGCTGTATGATAACCTGAGCTACGCAGAAGTTCTTGATAAAGAACTAAAAGTGATGGATTTAGCAGCATTTACACTGGCTCGTGACCAC----------

>Strain:3DA3 Gene:pyrH

---------------------------------------------------------------------------------------------------------------------------------------CTGGGTGTTCAGGTCGGTGTGGTTATCGGTGGTGGTAACTTGTTCCGTGGTGCAGGCCTTGCTGAAGCGGGTATGAACCGTGTTGTTGGTGACCACATGGGTATGTTAGCAACAGTAATGAACGGTTTAGCGATGCGTGACGCTTTGCACCGTGCATACGTCAATGCTCGTGTTATGTCTGCAATCCCACTAAAAGGTGTGTGTGACGATTACAACTGGGCAGATGCAATCCGTGAACTGCGCCAAGGCCGTGTTGTTATCTTCTCAGCAGGTACAGGCAACCCATTCTTCACAACAGATTCTGCTGCATGTCTACGTGGAATTGAAATTGAAGCTGACGTAGTTCTAAAAGCGACAAAAGTTGATGGTGTATTTACGGCTGACCCAGTAGCCAACCCTGACGCAGAGCTGTATGATAACCTGAGCTACGCAGAAGTTCTTGATAAAGAACTAAAAGTG----------------------------------------------

>Strain:3DA1 Gene:pyrH

----------------------------------------------------------------------------------------------------------------AAGAAGTAAAAGAACTGGTTGAACTGGGTGTTCAGGTCGGTGTGGTTATCGGTGGTGGTAACTTGTTCCGTGGTGCAGGCCTTGCTGAAGCGGGTATGAACCGTGTTGTTGGTGACCACATGGGTATGTTAGCAACAGTAATGAACGGTTTAGCGATGCGTGACGCTTTGCACCGTGCATACGTCAATGCTCGTGTTATGTCTGCAATCCCACTAAAAGGTGTGTGTGACGATTACAACTGGGCAGATGCAATCCGTGAACTGCGCCAAGGCCGTGTTGTTATCTTCTCAGCAGGTACAGGCAACCCATTCTTCACAACAGATTCTGCTGCATGTCTACGTGGAATTGAAATTGAAGCTGACGTAGTTCTAAAAGCGACAAAAGTTGATGGTGTATTTACGGCTGACCCAGTAGCCAACCCTGACGCAGAGCTGTATGATAACCTGAGCTACGCAGAAGTTCTTGATAAAGAACTAAAAGTGATGGATTTAGCAGCATTTACACTGGCTCGTGACC------------

>Strain:3DA2 Gene:pyrH

---------------------------------------------------------------------------------------------------------------CAAGAAGTAAAAGAACTGGTTGAACTGGGTGTTCAGGTCGGTGTGGTTATCGGTGGTGGTAACTTGTTCCGTGGTGCAGGCCTTGCTGAAGCGGGTATGAACCGTGTTGTTGGTGACCACATGGGTATGTTAGCAACAGTAATGAACGGTTTAGCGATGCGTGACGCTTTGCACCGTGCATACGTCAATGCTCGTGTTATGTCTGCAATCCCACTAAAAGGTGTGTGTGACGATTACAACTGGGCAGATGCAATCCGTGAACTGCGCCAAGGCCGTGTTGTTATCTTCTCAGCAGGTACAGGCAACCCATTCTTCACAACAGATTCTGCTGCATGTCTACGTGGAATTGAAATTGAAGCTGACGTAGTTCTAAAAGCGACAAAAGTTGATGGTGTATTTACGGCTGACCCAGTAGCCAACCCTGACGCAGAGCTGTATGATAACCTGAGCTACGCAGAAGTTCTTGATAAAGAACTAAAAGTGATGGATTTAGCAGCATTTACACTGGCTCGTGACC------------

>Strain:42B Gene:pyrH

------------------------------------------------------------------------------------------------------------GCTCAAGAAGTAAAAGAACTGGTTGAACTGGGTGTTCAGGTCGGTGTGGTTATCGGTGGTGGTAACTTGTTCCGTGGTGCAGGTCTTGCTGAAGCGGGTATGAACCGTGTTGTTGGTGACCACATGGGTATGTTAGCAACAGTAATGAACGGTTTGGCGATGCGTGATGCTTTGCACCGTGCATACGTCAATGCTCGTGTTATGTCTGCAATCCCACTGAAAGGCGTGTGTGACGATTACAACTGGGCAGATGCAATCCGCGAACTGCGCCAAGGCCGTGTTGTTATCTTCTCAGCAGGTACAGGTAACCCATTCTTCACAACTGATTCTGCTGCATGTCTACGCGGAATTGAAATTGAAGCTGACGTAGTTCTAAAAGCGACAAAAGTTGATGGTGTATTTACGGCTGACCCAGTAGCCAACCCTGACGCAGAGCTGTATGATAACCTGAGCTACGCAGAAGTTCTTGATAAAGAACTAAAAGTGATGGATTTAGCAGCATTTACACTGGCTCGTGACCAC----------

>Strain:43C Gene:pyrH

-----------------------------------------------------------------------------------------------------------------AGAAGTAAAAGAACTGGTTGAACTGGGTGTTCAGGTCGGTGTGGTTATCGGTGGTGGTAACTTGTTCCGTGGTGCAGGTCTTGCTGAAGCGGGTATGAACCGTGTTGTTGGTGACCACATGGGTATGTTAGCAACAGTAATGAACGGTTTGGCGATGCGTGATGCTTTGCACCGTGCATACGTCAATGCTCGTGTTATGTCTGCAATCCCACTAAAAGGTGTGTGTGACGATTACAACTGGGCAGACGCAATCCGTGAGTTGCGCCAAAGCCGTGTTGTTATCTTCTCAGCAGGTACAGGTAACCCATTCTTCACAACCGATTCTGCTGCATGTCTACGCGGAATTGAAATTGAAGCTGACGTAGTTCTAAAAGCGACAAAAGTTGATGGTGTATTTACGGCTGACCCAGTAGCCAACCCTGACGCAGAGCTGTATGATAACCTGAGCTACGCAGAAGTTCTTGATAAAGAACTAAAAGTGATGGATTTAGCAGCATTTACACTGGCTCG-----------------

>Strain:43O Gene:pyrH

----------------------------------------------------------------------------------------------------------------AAGAAGTAAAAGAACTGGTTGAACTGGGTGTTCAGGTCGGTGTGGTTATCGGTGGTGGTAACTTGTTCCGTGGTGCAGGTCTTGCTGAAGCGGGTATGAACCGTGTTGTTGGTGACCACATGGGTATGTTAGCAACAGTAATGAACGGTTTGGCGATGCGTGATGCTTTGCACCGTGCATACGTCAATGCTCGTGTTATGTCTGCAATCCCACTGAAAGGTGTGTGTGACGATTACAACTGGGCAGATGCAATCCGCGAACTGCGCCAAGGTCGTGTTGTTATCTTCTCAGCAGGTACAGGTAACCCATTCTTCACAACCGATTCTGCTGCATGTCTACGCGGAATTGAAATTGAAGCTGACGTAGTTCTAAAAGCGACAAAAGTTGATGGTGTATTTACGGCTGACCCAGTAGCCAACCCTGACGCAGAGCTGTATGATAACCTGAGCTACGCAGAAGTTCTTGATAAAGAACTAAAAGTGATGGATTTAGCAGCATTTACACTGGCTCGTGAC-------------

>Strain:43P Gene:pyrH

-------------------------------------------------------------------------------------------------------------CTCAAGAAGTAAAAGAACTGGTTGAACTGGGTGTTCAGGTCGGTGTGGTTATCGGTGGTGGTAACTTGTTCCGTGGTGCAGGTCTTGCTGAAGCGGGTATGAACCGTGTTGTTGGTGACCACATGGGTATGTTAGCAACAGTAATGAACGGTTTGGCGATGCGTGATGCTTTGCACCGTGCATACGTCAATGCTCGTGTTATGTCTGCAATCCCACTGAAAGGTGTGTGTGACGATTACAACTGGGCAGATGCAATCCGCGAACTGCGCCAAGGTCGTGTTGTTATCTTCTCAGCAGGTACAGGTAACCCATTCTTCACAACCGATTCTGCTGCATGTCTACGCGGAATTGAAATTGAAGCTGACGTAGTTCTAAAAGCGACAAAAGTTGATGGTGTATTTACGGCTGACCCAGTAGCCAACCCTGACGCAGAGCTGTATGATAACCTGAGCTACGCAGAAGTTCTTGATAAAGAACTAAAAGTGATGGATTTAGCAGCATTTACACTGGCTCGTGACCACAAAATGCCTA

>Strain:43Q Gene:pyrH

----------------------------------------------------------------------------------------------------------------AAGAAGTAAAAGAACTGGTTGAACTGGGTGTTCAGGTCGGTGTGGTTATCGGTGGTGGTAACTTGTTCCGTGGTGCAGGTCTTGCTGAAGCGGGTATGAACCGTGTTGTTGGTGACCACATGGGTATGTTAGCAACAGTAATGAACGGTTTGGCGATGCGTGATGCTTTGCACCGTGCATACGTCAATGCTCGTGTTATGTCTGCAATCCCACTGAAAGGTGTGTGTGACGATTACAACTGGGCAGATGCAATCCGCGAACTGCGCCAAGGTCGTGTTGTTATCTTCTCAGCAGGTACAGGTAACCCATTCTTCACAACCGATTCTGCTGCATGTCTACGCGGAATTGAAATTGAAGCTGACGTAGTTCTAAAAGCGACAAAAGTTGATGGTGTATTTACGGCTGACCCAGTAGCCAACCCTGACGCAGAGCTGTATGATAACCTGAGCTACGCAGAAGTTCTTGATAAAGAACTAA---------------------------------------------------

>Strain:45B Gene:pyrH

------------------------------------------------------------------------------------------------------------GCTCAAGAAGTAAAAGAACTGGTTGAACTGGGTGTTCAGGTCGGTGTGGTTATCGGTGGTGGTAACTTGTTCCGTGGTGCAGGTCTTGCTGAAGCGGGTATGAACCGTGTTGTTGGTGACCACATGGGTATGTTAGCAACAGTAATGAACGGTTTGGCGATGCGTGATGCTTTGCACCGTGCATACGTCAATGCTCGTGTTATGTCTGCAATCCCACTGAAAGGTGTGTGTGACGATTACAACTGGGCAGATGCAATCCGCGAACTGCGCCAAGGCCGTGTTGTTATCTTCTCAGCAGGTACAGGTAACCCATTCTTCACAACCGATTCTGCTGCATGTCTACGCGGAATTGAAATTGAAGCTGACGTAGTTCTAAAAGCGACAAAAGTTGATGGTGTATTTACGGCTGACCCAGTAGCCAACCCTGACGCAGAGCTGTATGATAACCTGAGCTACGCAGAAGTTCTTGATAAAGAACTAAAAGTGATGGATTTAGCAGCATTTACACTGGCTCGTGACCA-----------

>Strain:45C Gene:pyrH

--------------------------------------------------------------------------------------------------------------TCAAGAAGTAAAAGAACTGGTTGAACTGGGTGTTCAGGTCGGTGTGGTTATCGGTGGTGGTAACTTGTTCCGTGGTGCAGGTCTTGCTGAAGCGGGTATGAACCGTGTTGTTGGTGACCACATGGGTATGTTAGCAACAGTAATGAACGGTTTGGCGATGCGTGATGCTTTGCACCGTGCATACGTCAATGCTCGTGTTATGTCTGCAATCCCACTAAAAGGTGTGTGTGACGATTACAACTGGGCAGATGCAATCCGCGAGTTGCGCCAAGGCCGTGTTGTTATCTTCTCAGCAGGTACAGGTAACCCATTCTTCACAACAGATTCTGCTGCATGCCTACGCGGAATTGAAATTGAAGCTGACGTAGTTCTAAAAGCGACAAAAGTTGATGGTGTATTTACGGCTGACCCAGTAGCCAACCCTGACGCAGAGCTGTATGATAACCTGAGCTACGCAGAAGTTCTTGATAAAGAACTAAAAGTGATGGATTTAGCAGCATTTACACTGGCTCGTGACCA-----------

>Strain:45D Gene:pyrH

----------------------------------------------------------------------------------------------------------------AAGAAGTAAAAGAACTGGTTGAACTGGGTGTTCAGGTCGGTGTGGTTATCGGTGGTGGTAACTTGTTCCGTGGTGCAGGTCTTGCTGAAGCGGGTATGAACCGTGTTGTTGGTGACCACATGGGTATGTTAGCAACAGTAATGAACGGTTTGGCGATGCGTGATGCTTTGCACCGTGCATACGTCAATGCTCGTGTTATGTCTGCAATCCCACTGAAAGGTGTGTGTGACGATTACAACTGGGCAGATGCAATCCGCGAACTGCGCCAAGGCCGTGTTGTTATCTTCTCAGCAGGTACAGGTAACCCATTCTTCACAACCGATTCTGCTGCATGTCTACGCGGAATTGAAATTGAAGCTGACGTAGTTCTAAAAGCGACAAAAGTTGATGGTGTATTTACGGCTGACCCAGTAGCCAACCCTGACGCAGAGCTGTATGATAACCTGAGCTACGCAGAAGTTCTTGATAAAGAACTAAAAGTGATGGATTTAGCAGCATTTACACTGGCTCGTGA--------------

>Strain:LMG 19270 Gene:pyrH

GGACGAAATCCTAAACCAGCGTATCAACGTATTCTGTTAAAACTGAGTGGTGAAGCACTTCAAGGCGAAGAAGGTTTTGGTATTGATCCTGCGATCTTGGATCGTATGGCTCAAGAAGTAAAAGAACTGGTTGAACTGGGTGTTCAGGTCGGTGTGGTTATCGGTGGTGGTAACTTGTTCCGTGGTGCAGGTCTTGCTGAAGCGGGTATGAACCGTGTTGTTGGTGACCACATGGGTATGTTAGCAACAGTAATGAATGGTTTGGCGATGCGTGATGCTTTGCACCGTGCATACGTCAATGCTCGTGTTATGTCTGCAATCCCACTAAAAGGTGTGTGTGACGATTACAACTGGGCAGACGCCATCCGCGAGTTGCGCCAAGGCCGTGTTGTTATCTTCTCAGCAGGTACAGGTAACCCATTCTTCACAACCGATTCTGCTGCATGTCTACGCGGAATTGAAATTGAAGCTGACGTAGTTCTAAAAGCGACAAAAGTTGATGGTGTATTTACGGCTGACCCAGTAGCCAACCCTGACGCAGAGCTGTATGATAACCTGAGCTACGCAGAAGTTCTTGATAAAGAACTAAAAGTGATGGA-----------------------------------------

>Strain:R-621 Gene:pyrH

-------------------------------------------------------------------------------------------------------------------AAGTAAAAGAGCTGGTTGAACTGGGTGTTCAAGTTGGTGTGGTAATCGGTGGTGGTAACCTGTTCCGTGGCGCTGGTCTTGCGGAAGCTGGTATGAACCGCGTAGTAGGCGACCACATGGGTATGCTTGCAACGGTAATGAACGGTTTGGCAATGCGTGACGCACTTCACCGTGCATACGTAAACGCTCGTGTAATGTCTGCAATTCCTCTAAAAGGTGTGTGTGACGACTACAATTGGGCAGACGCTATCCGCGAACTTCGTCAAGGCCGAGTGGTAATCTTCTCTGCAGGTACTGGTAACCCATTCTTCACAACAGATTCAGCGGCGTGTCTACGTGGTATCGAAATTGAAGCTGACGTAGTTCTAAAAGCAACGAAAGTTGATGGCGTATTTACTGCAGACCCAGTAGCAAACCCAGACGCAGAGCTGTATGAT----------------------------------------------------------------------------------------

>Strain:R-41 Gene:pyrH

---------------------------------------------------------------------------------------------------------------------------------GTTGAACTGGGTGTTCAAGTTGGTGTGGTAATCGGTGGTGGTAACCTGTTCCGTGGCGCTGGTCTTGCGGAAGCTGGTATGAACCGCGTAGTAGGCGACCACATGGGTATGCTTGCAACGGTAATGAACGGTTTGGCAATGCGTGACGCACTTCACCGTGCATACGTAAACGCTCGTGTAATGTCTGCAATTCCTCTAAAAGGTGTGTGTGACGACTACAATTGGGCAGACGCTATCCGCGAACTTCGTCAAGGCCGAGTGGTAATCTTCTCTGCAGGTACTGGTAACCCATTCTTCACAACAGATTCAGCGGCGTGTCTACGTGGTATCGAAATTGAAGCTGACGTAGTTCTAAAAGCAACGAAAGTTGATGGCGTATTTACTGCAGACCCAGTAGCAAACCCAGACGCAGAGCTGTATGAT----------------------------------------------------------------------------------------

>Strain:R-718 Gene:pyrH

---------------------------------------------------------------------------------------------------------------------GTTAAAGAGCTGNTTGAACTGGGTGTTCAAGTTGGTGTGGTAATCGGTGGTGGTAACCTGTTCCGTGGCGCTGGTCTTGCGGAAGCTGGTATGAACCGCGTAGTAGGCGACCACATGGGTATGCTTGCAACGGTAATGAACGGTTTGGCAATGCGTGACGCACTTCACCGTGCATACGTAAACGCTCGTGTAATGTCTGCAATTCCTCTAAAAGGTGTGTGTGACGACTACAATTGGGCAGACGCTATCCGCGAACTTCGTCAAGGCCGAGTGGTAATCTTCTCTGCAGGTACTGGTAACCCATTCTTCACAACAGATTCAGCGGCGTGTCTACGTGGTATCGAAATTGAAGCTGACGTAGTTCTTAAAGCAACGAAAGTTGATGGCGTATTTACTGCAGACCCAGTAGCAAACCCAGACGCAGAGCTGTATGAT----------------------------------------------------------------------------------------

>Strain:R-694 Gene:pyrH

----------------------------------------------------------------------------------------------------------------AAGAAGTTAAAGAGCTGGTTGAACTGGGTGTTCAAGTTGGTGTGGTAATCGGTGGTGGTAACCTGTTCCGTGGCGCTGGTCTTGCGGAAGCTGGTATGAACCGCGTAGTAGGCGACCACATGGGTATGCTTGCAACGGTAATGAACGGTTTGGCAATGCGTGACGCACTTCACCGTGCATACGTAAACGCTCGTGTAATGTCTGCAATTCCTCTAAAAGGTGTGTGTGACGACTACAATTGGGCAGACGCTATCCGCGAACTTCGTCAAGGCCGAGTGGTAATCTTCTCTGCAGGTACTGGTAACCCATTCTTCACAACAGATTCAGCGGCGTGTCTACGTGGTATCGAAATTGAAGCTGACGTAGTTCTAAAAGCAACGAAAGTTGATGGCGTATTTACTGCAGACCCAGTAGCAAACCCAGACGCAGAGCTGTATGAT----------------------------------------------------------------------------------------

>Strain:R-688 Gene:pyrH

----------------------------------------------------------------------------------------------------------------AAGAAGTTAAAGAGCTGGTTGAACTGGGTGTTCAAGTTGGTGTGGTAATCGGTGGTGGTAACCTGTTCCGTGGCGCTGGTCTTGCGGAAGCTGGTATGAACCGCGTAGTAGGCGACCACATGGGTATGCTTGCAACGGTAATGAACGGTTTGGCAATGCGTGACGCACTTCACCGTGCATACGTAAACGCTCGTGTAATGTCTGCAATTCCTCTAAAAGGTGTGTGTGACGACTACAATTGGGCAGACGCTATCCGCGAACTTCGTCAAGGCCGAGTGGTAATCTTCTCTGCAGGTACTGGTAACCCATTCTTCACAACAGATTCAGCGGCGTGTCTACGTGGTATCGAAATTGAAGCTGACGTAGTTCTAAAAGCAACGAAAGTTGATGGCGTATTTACTGCAGACCCAGTAGCAAACCCAGACGCAGAGCTGTATGAT----------------------------------------------------------------------------------------

>Strain:R-662 Gene:pyrH

------------------------------------------------------------------------------------------------------------------AGAAGTAAAGAGCTGGTTGAACTGGGTGTTCAAGTTGGTGTGGTAATCGGTGGTGGTAACCTGTTCCGTGGCGCTGGTCTTGCGGAAGCTGGTATGAACCGCGTAGTAGGCGACCACATGGGTATGCTTGCAACGGTAATGAACGGTTTGGCAATGCGTGACGCACTTCACCGTGCATACGTAAACGCTCGTGTAATGTCTGCAATTCCTCTAAAAGGTGTGTGTGACGACTACAATTGGGCAGACGCTATCCGCGAACTTCGTCAAGGCCGAGTGGTAATCTTCTCTGCAGGTACTGGTAACCCATTCTTCACAACAGATTCAGCGGCGTGTCTACGTGGTATCGAAATTGAAGCTGACGTAGTTCTAAAAGCAACGAAAGTTGATGGCGTATTTACTGCAGACCCAGTAGCAAACCCAGACGCAGAGCTGTATGAT----------------------------------------------------------------------------------------

>Strain:R-690 Gene:pyrH

------------------------------------------------------------------------------------------------------------------GAAGTTAAAGAGCTGGTTGAACTGGGTGTTCAAGTTGGTGTGGTAATCGGTGGTGGTAACCTGTTCCGTGGCGCTGGTCTTGCGGAAGCTGGTATGAACCGCGTAGTAGGCGACCACATGGGTATGCTTGCAACGGTAATGAACGGTTTGGCAATGCGTGACGCACTTCACCGTGCATACGTAAACGCTCGTGTAATGTCTGCAATTCCTCTAAAAGGTGTGTGTGACGACTACAATTGGGCAGACGCTATCCGCGAACTTCGTCAAGGCCGAGTGGTAATCTTCTCTGCAGGTACTGGTAACCCATTCTTCACAACAGATTCAGCGGCGTGTCTACGTGGTATCGAAATTGAAGCTGACGTAGTTCTAAAAGCAACGAAAGTTGATGGCGTATTTACTGCAGACCCAGTAGCAAACCCAGACGCAGAGCTGTATGAT----------------------------------------------------------------------------------------
